# Supplementary material for: Scoping review of the literature on outcomes of the conservation reserve program
Source: PLoS One. 2025 Aug 12;20(8):e0329962. doi: 10.1371/journal.pone.0329962 (PMC12342260; doi:10.1371/journal.pone.0329962)
Supplement: S2 Table — (DOCX) [file pone.0329962.s002.docx]

Table S1: Table comparing CRP acreage to number of CRP studies occurring in each U.S. state. Table is ordered by CRP acreage for all 50 states. Rank CRP Studies indicates the rank of states based on the total number of CRP studies with 1 having the most studies and 39 having the least. When states were tied for number of studies, they were all given the same rank number and the proceeding state received the next number when accounting for the number of tied states (e.g., if 2 states were ranked 3 the following state would be ranked 5). NAs in columns for CRP Acreage and Rank CRP Studies respectively indicate that there is no CRP acreage in that state and that no peer-reviewed CRP studies were found for that state. CRP acreage numbers were collected from https://www.fsa.usda.gov/Assets/USDA-FSA-Public/usdafiles/Conservation/PDF/CRP%20Years%20Enrolled%20by%20State%20Sep%202017.pdf

| **State** | **CRP Acreage** | **CRP Studies** | **Rank CRP Studies** |
| --- | --- | --- | --- |
| Texas | 2,896,417 | 54 | 5 |
| Kansas | 2,071,558 | 87 | 1 |
| Iowa | 1,786,516 | 69 | 3 |
| Colorado | 1,779,117 | 40 | 11 |
| North Dakota | 1,528,588 | 72 | 2 |
| Montana | 1,364,108 | 35 | 12 |
| Washington | 1,195,638 | 26 | 16 |
| Minnesota | 1,128,128 | 53 | 6 |
| South Dakota | 976,938 | 53 | 6 |
| Missouri | 965,462 | 52 | 8 |
| Illinois | 895,409 | 43 | 9 |
| Nebraska | 800,378 | 56 | 4 |
| Mississippi | 699,502 | 27 | 15 |
| Oklahoma | 685,344 | 42 | 10 |
| Idaho | 561,592 | 17 | 19 |
| Oregon | 485,809 | 9 | 23 |
| New Mexico | 446,902 | 20 | 18 |
| Louisiana | 287,148 | 5 | 29 |
| Ohio | 257,519 | 17 | 19 |
| Kentucky | 248,794 | 8 | 27 |
| Alabama | 241,962 | 9 | 23 |
| Georgia | 238,538 | 11 | 21 |
| Indiana | 231,299 | 22 | 17 |
| Wisconsin | 231,276 | 28 | 14 |
| Arkansas | 230,272 | 9 | 23 |
| Wyoming | 190,440 | 9 | 23 |
| Utah | 165,019 | 5 | 29 |
| Pennsylvania | 150,846 | 5 | 29 |
| Tennessee | 137,748 | 10 | 22 |
| Michigan | 127,155 | 33 | 13 |
| South Carolina | 77,101 | 2 | 35 |
| California | 74,337 | 1 | 39 |
| North Carolina | 66,666 | 2 | 35 |
| Maryland | 58,239 | 8 | 27 |
| Virginia | 44,736 | 4 | 32 |
| New York | 34,177 | 2 | 35 |
| Florida | 29,841 | 2 | 35 |
| Alaska | 17,439 | 4 | 32 |
| Maine | 7,744 | 1 | 39 |
| West Virginia | 6,357 | 1 | 39 |
| Delaware | 4,100 | 3 | 34 |
| Vermont | 2,720 | 1 | 39 |
| New Jersey | 2,049 | 1 | 39 |
| Hawaii | 1,168 | 0 | NA |
| Nevada | 146 | 0 | NA |
| Connecticut | 59 | 1 | 39 |
| Rhode Island | 28 | 1 | 39 |
| Massachusetts | 10 | 1 | 39 |
| New Hampshire | 4 | 1 | 39 |
| Arizona | NA | 0 | NA |
