# Supplementary material for: Scoping review of the literature on outcomes of the conservation reserve program
Source: PLoS One. 2025 Aug 12;20(8):e0329962. doi: 10.1371/journal.pone.0329962 (PMC12342260; doi:10.1371/journal.pone.0329962)
Supplement: Attachment — Submitted filename: PONE-D-24-54508_MArkedUP.pdf [file pone.0329962.s005.pdf]

PLOS ONE

Scoping Review and synthesis of the literature on the outcomes of the Conservation Reserve Program

--Manuscript Draft--

**Manuscript Number:**	PONE-D-24-54508
**Article Type:**	Research Article
**Full Title:**	Scoping Review and synthesis of the literature on the outcomes of the Conservation Reserve Program
**Short Title:**	Conservation Reserve Program: Scoping Review
**Corresponding Author:**	Mark Nessel<br>Oklahoma State University Stillwater College of Agricultural Sciences and Natural Resources: Oklahoma State University Ferguson College of Agriculture<br>Stillwater, Oklahoma UNITED STATES OF AMERICA
**Keywords:**	Conservation Reserve Program; biodiversity; Ecosystem Services; Land Restoration
**Abstract:**	The Conservation Reserve Program (CRP), one of the largest private lands conservation programs in the U.S., has played a critical role in soil erosion reduction, water quality improvement, wildlife habitat enhancement, and carbon sequestration since its inception in 1985. Despite extensive research illustrating these many benefits of the program, a synthetic, broad-scale view of the literature on CRP would allow identification of emphases, gaps, and trends in this literature and could inform future research and funding decisions related to this critical program. We addressed this need by conducting a scoping review of the peer-reviewed CRP literature. Through systematic analysis of 577 studies, we examined spatial and temporal trends in the literature, categorized CRP research into five major categories—including studies of wildlife, vegetation, air/soil/water, productivity, and social aspects—and conducted a detailed evaluation of the outcomes of CRP that have been studied for wildlife and vegetation. Studies of wildlife-related outcomes of CRP, and particularly studies of birds, dominate the literature, while research on other taxa such as fish, reptiles, and amphibians remains sparse. Geographically, most studies are concentrated in the Great Plains, leaving regions such as the Pacific Northwest underrepresented relative to CRP land share. This review highlights the need for long-term studies and additional research, including less-studied taxa and underrepresented regions, to better understand CRP's potential for enhancing biodiversity and ecosystem services. These findings provide a foundation for future research and policy decisions, ensuring the CRP's continued contribution to land conservation across diverse ecosystems.
**Order of Authors:**	Mark Nessel
	Karen Maguire
	Rich Iovanna
	Courtney J Duchardt
	Scott R. Loss
**Additional Information:**	
**Question**	**Response**
**Financial Disclosure**<br><br>Enter a financial disclosure statement that describes the sources of funding for the work included in this submission. Review the submission guidelines for detailed requirements. View published research articles from *PLOS ONE* for specific examples.	Yes

Powered by Editorial Manager® and ProduXion Manager® from Aries Systems Corporation

This statement is required for submission and **will appear in the published article** if the submission is accepted. Please make sure it is accurate.<br><br>**Funded studies**<br>Enter a statement with the following details:<br>• Initials of the authors who received each award<br>• Grant numbers awarded to each author<br>• The full name of each funder<br>• URL of each funder website<br>• Did the sponsors or funders play any role in the study design, data collection and analysis, decision to publish, or preparation of the manuscript?<br><br>Did you receive funding for this work?	
Please add funding details.<br>as follow-up to "**Financial Disclosure**<br><br>Enter a financial disclosure statement that describes the sources of funding for the work included in this submission. Review the submission guidelines for detailed requirements. View published research articles from *PLOS ONE* for specific examples.<br><br>This statement is required for submission and **will appear in the published article** if the submission is accepted. Please make sure it is accurate.<br><br>**Funded studies**<br>Enter a statement with the following details:<br>• Initials of the authors who received each award<br>• Grant numbers awarded to each author<br>• The full name of each funder<br>• URL of each funder website<br>• Did the sponsors or funders play any role in the study design, data collection and analysis, decision to publish, or preparation of the manuscript?	This research was funded by the U.S. Department of Agriculture (USDA) Natural Resources Conservation Service (Award # NR233A750023C009), the USDA Economic Research Service (Agreement #58-6000-2-0071), and USDA Hatch Grant funds through the Oklahoma Agricultural Experiment Station (OKL03231 and OKL02915). We thank all authors whose work was included in our scoping review. The findings and conclusions in this presentation are those of the author(s) and should not be construed to represent any official USDA or U.S. Government determination or policy.

Powered by Editorial Manager® and ProduXion Manager® from Aries Systems Corporation

Did you receive funding for this work?"	
Please select the country of your main research funder (please select carefully as in some cases this is used in fee calculation).<br>as follow-up to "**Financial Disclosure**<br><br>Enter a financial disclosure statement that describes the sources of funding for the work included in this submission. Review the submission guidelines for detailed requirements. View published research articles from *PLOS ONE* for specific examples.<br><br>This statement is required for submission and **will appear in the published article** if the submission is accepted. Please make sure it is accurate.<br><br>**Funded studies**<br>Enter a statement with the following details:<br>• Initials of the authors who received each award<br>• Grant numbers awarded to each author<br>• The full name of each funder<br>• URL of each funder website<br>• Did the sponsors or funders play any role in the study design, data collection and analysis, decision to publish, or preparation of the manuscript?<br><br>Did you receive funding for this work?"	UNITED STATES - US
**Competing Interests**<br><br>Use the instructions below to enter a competing interest statement for this submission. On behalf of all authors, disclose any competing interests that could be perceived to bias this work—acknowledging all financial support and any other relevant financial or non-financial competing interests.<br><br>This statement is required for submission	The authors have declared that no competing interests exist.

and **will appear in the published article** if the submission is accepted. Please make sure it is accurate and that any funding sources listed in your Funding Information later in the submission form are also declared in your Financial Disclosure statement.<br><br>View published research articles from *PLOS ONE* for specific examples.<br><br>**NO authors have competing interests**<br><br>Enter: *The authors have declared that no competing interests exist.*<br><br>**Authors with competing interests**<br><br>Enter competing interest details beginning with this statement:<br><br>*I have read the journal's policy and the authors of this manuscript have the following competing interests: [insert competing interests here]*<br><br>* typeset	
**Ethics Statement**<br><br>Enter an ethics statement for this submission. This statement is required if the study involved:<br><br>• Human participants<br>• Human specimens or tissue<br>• Vertebrate animals or cephalopods<br>• Vertebrate embryos or tissues<br>• Field research<br><br>Write "N/A" if the submission does not require an ethics statement.<br><br>General guidance is provided below. Consult the submission guidelines for detailed instructions. **Make sure that all information entered here is included in the Methods section of the manuscript.**	N/A

**Format for specific study types**<br><br>**Human Subject Research (involving human participants and/or tissue)**<br>• Give the name of the institutional review board or ethics committee that approved the study<br>• Include the approval number and/or a statement indicating approval of this research<br>• Indicate the form of consent obtained (written/oral) or the reason that consent was not obtained (e.g. the data were analyzed anonymously)<br><br>**Animal Research (involving vertebrate animals, embryos or tissues)**<br>• Provide the name of the Institutional Animal Care and Use Committee (IACUC) or other relevant ethics board that reviewed the study protocol, and indicate whether they approved this research or granted a formal waiver of ethical approval<br>• Include an approval number if one was obtained<br>• If the study involved *non-human primates*, add *additional details* about animal welfare and steps taken to ameliorate suffering<br>• If anesthesia, euthanasia, or any kind of animal sacrifice is part of the study, include briefly which substances and/or methods were applied<br><br>**Field Research**<br><br>Include the following details if this study involves the collection of plant, animal, or other materials from a natural setting:<br>• Field permit number<br>• Name of the institution or relevant body that granted permission	
**Data Availability**<br><br>Authors are required to make all data underlying the findings described fully available, without restriction, and from the time of publication. PLOS allows rare exceptions to address legal and ethical concerns. See the PLOS Data Policy and FAQ for detailed information.	Yes - all data are fully available without restriction

Powered by Editorial Manager® and ProduXion Manager® from Aries Systems Corporation

A Data Availability Statement describing where the data can be found is required at submission. Your answers to this question constitute the Data Availability Statement and **will be published in the article**, if accepted.<br><br>**Important:** Stating 'data available on request from the author' is not sufficient. If your data are only available upon request, select 'No' for the first question and explain your exceptional situation in the text box.<br><br>Do the authors confirm that all data underlying the findings described in their manuscript are fully available without restriction?	
**Describe where the data may be found in full sentences. If you are copying our sample text, replace any instances of XXX with the appropriate details.**<br><br>• If the data are **held or will be held in a public repository**, include URLs, accession numbers or DOIs. If this information will only be available after acceptance, indicate this by ticking the box below. For example: *All XXX files are available from the XXX database (accession number(s) XXX, XXX.).*<br>• If the data are all contained **within the manuscript and/or Supporting Information files**, enter the following: *All relevant data are within the manuscript and its Supporting Information files.*<br>• If neither of these applies but you are able to provide **details of access elsewhere**, with or without limitations, please do so. For example:<br><br>*Data cannot be shared publicly because of [XXX]. Data are available from the XXX Institutional Data Access / Ethics Committee (contact via XXX) for researchers who meet the criteria for access to confidential data.*<br><br>*The data underlying the results presented in the study are available from (include the name of the third party*	All relevant data are within the manuscript and its Supporting Information files

and contact information or URL).<br>• This text is appropriate if the data are owned by a third party and authors do not have permission to share the data.<br><br>* typeset	
Additional data availability information:	

Manuscript Click here to access/download;Manuscript;Manuscript.docx

Scoping Review and synthesis of the literature on the outcomes of the Conservation Reserve Program.

Running Title: Conservation Reserve Program: Scoping Review

Authors: Mark P. Nessel[1*], Karen Maguire[2], Rich Iovanna[3], Courtney J Duchardt[1,4], Scott R. Loss [1]

[1] Department of Natural Resource Ecology and Management, Oklahoma State University, Stillwater, OK

[2] USDA Economic Research Service, U.S. Department of Agriculture, Kansas City, MO

[3] USDA Farm Production and Conservation Mission Area, Economic and Policy Analysis Division, Washington, DC

[4] School of Natural Resources and the Environment, University of Arizona, Tucson, AZ

*Corresponding author: nesselm13@gmail.com

Keywords:

Conservation Reserve Program, Biodiversity, Ecosystem Services, Land Restoration

Abstract

The Conservation Reserve Program (CRP), one of the largest private lands conservation programs in the U.S., has played a critical role in soil erosion reduction, water quality improvement, wildlife habitat enhancement, and carbon sequestration since its inception in 1985. Despite extensive research illustrating these many benefits of the program, a synthetic, broad-scale view of the literature on CRP would allow identification of emphases, gaps, and trends in this literature and could inform future research and funding decisions related to this critical program. We addressed this need by conducting a scoping review of the peer-reviewed CRP literature. Through systematic analysis of 577 studies, we examined spatial and temporal trends in the literature, categorized CRP research into five major categories—including studies of wildlife, vegetation, air/soil/water, productivity, and social aspects—and conducted a detailed evaluation of the outcomes of CRP that have been studied for wildlife and vegetation. Studies of wildlife-related outcomes of CRP, and particularly studies of birds, dominate the literature, while research on other taxa such as fish, reptiles, and amphibians remains sparse. Geographically, most studies are concentrated in the Great Plains, leaving regions such as the Pacific Northwest underrepresented relative to CRP land share. This review highlights the need for long-term studies and additional research, including less-studied taxa and underrepresented regions, to better understand CRP's potential for enhancing biodiversity and ecosystem services. These findings provide a foundation for future research and policy decisions, ensuring the CRP's continued contribution to land conservation across diverse ecosystems.

Introduction

Agriculture production is crucial for sustaining human livelihoods and societies and is also one of the most substantial ways humans alter the earth's land cover. The Food and Agriculture Organization of the United Nations estimates that agricultural land makes up 48 million km^2, or about 45% of all habitable land on earth (FAO 2024; Ritchie & Roser 2019). Land use changes are contributing substantially to biodiversity loss (Caro *et al.* 2022; Jaureguiberry *et al.* 2022). Government programs designed to set aside marginal or abandoned farmland for purposes of land restoration, resource renewal, and biodiversity conservation (Baylis *et al.* 2022; Bryan *et al.* 2018; Chazdon *et al.* 2020; Fayet *et al.* 2022) exist across the globe, but these programs are subject to changes in economic and political conditions, and many have only lasted for a short time without being renewed (Baylis *et al.* 2022; Kong *et al.* 2023).

One of the largest and longest-standing private-lands conservation programs is the U.S. Department of Agriculture (USDA) Farm Service Agency (FSA) Conservation Reserve Program (CRP). The CRP is a voluntary land conservation program that was established in 1985 with the primary goals of reducing soil erosion on environmentally sensitive cropland in the United States (USDA-FSA 2020). Subsequently, the goals have expanded to include improving water quality, providing wildlife habitat, and climate change mitigation (USDA-FSA 2020). For participation, landowners receive cost-share assistance for establishing approved land cover and annual rental payments for the duration of their CRP contract. Payments may also include incentive payments at sign-up or for work needed to maintain approved land cover on CRP lands. While annual enrollment fluctuates, CRP enrollment peaked in 2007 at 36.8 million acres, and in its first 35 years of existence (1986-2020) the program averaged 29.4 million acres enrolled annually (USDA-FSA 2020). Sharing a common theme of perennial cover, the broad range of management practices now supported by the program encompass a wide variety of vegetation

Spell out what CP (Conservation Practice) means for international audiences when first mentioned

types and conservation goals, including "Establishment of Permanent Native Grasses" (CP2), "Riparian Buffers" (CP22), and "Habitat Buffers for Upland Birds" (CP33) (USDA-FSA 2024).

Numerous studies have previously demonstrated a range of environmental benefits provided directly or indirectly from CRP. These benefits include fostering wildlife habitat (e.g., Herkert, 2007; Tanner et al., 2021), increasing abundance and productivity of beneficial insects (Begosh *et al.* 2022; Quinlan *et al.* 2023), fostering floral diversity (Begosh *et al.* 2022; Quinlan *et al.* 2021), mitigating flood risk (e.g., K. A. Johnson et al., 2016), restoring hydrology and reducing nutrient runoff (Lizotte *et al.* 2017; Locke *et al.* 2020), enhancing groundwater recharge (e.g., Rao & Yang, 2010), reducing soil erosion (e.g., Sullivan et al., 2004), and storing carbon and reducing greenhouse gas emissions (e.g., Gelfand et al., 2011; Moore et al., 2023). Beyond these environmental benefits, CRP also provides consistent annual rent payment to farmers, which may benefit producers during periods of market decline or who seek to dampen the effects of price and yield variability (Hodur *et al.* 2002; Wu & Weber 2012).

Limited time and resources mean that most ecological studies of the environmental benefits of CRP have been conducted by a single team of researchers working in a local study area, often for only a few years. However, CRP contract goals and implementation vary across the diverse ecosystems of the United States. Examining and describing CRP benefits broadly, across types of CRP and regions, may help inform potential program participants and policymakers about the benefits of CRP for land conservation. A more synthetic, broad-scale view of CRP allows us to identify trends across taxa, regions, ecosystem types, and CRP types, and to identify gaps in research to date.

Here, we conduct the first scoping review of peer-reviewed CRP research. A national-level systematic scoping review allows us to identify major research emphases and gaps in the

literature. Further, we discuss the specific outcomes, regions, taxa and CRP program characteristics that have been well-studied and highlight those that may require further research. Specifically, we systematically evaluated CRP literature to address three main objectives: 1) characterize spatial and temporal trends in CRP research, 2) categorize CRP research by type, including studies of wildlife, vegetation, soil and water, and more human-focused aspects (e.g., economic benefits and stakeholder perceptions of CRP), and 3) evaluate detailed characteristics of studies that focus on responses of wildlife and vegetation to CRP. With this review, we seek to inform and guide future research on important aspects of CRP that have to this point not been emphasized in the literature.

Methods

Search & exclusion criteria

We searched scientific literature sources for all studies that pertained to the Conservation Reserve Program. Using the search term "Conservation Reserve Program" (search conducted on February 15, 2024) we collected all studies on Web of Science (n = 966) and Scopus (n = 940). The two searches were combined, and duplicate studies were removed (n = 1167 unique studies; Table S1). Our review focused on studies specifically examining CRP outcomes and responses, as well as perceptions and behaviors related to CRP (hereafter "CRP studies"). Studies could have directly compared CRP to other land cover types, compared different CRP practices, or measured a response that included CRP as a factor. Our search included only peer-reviewed journal articles and excluded government reports that were not indexed in Web of Science or Scopus, article types that are either not peer-reviewed or were unlikely to contain original information about effects of CRP (i.e., editorial materials, meeting abstracts, theses,

commentaries). Further, in our preliminary search we found we were not able to comprehensively capture such non-peer-reviewed sources using our databases. Using broader sources like Google Scholar did allow us to capture non-peer-reviewed literature, but also exponentially increased the number of sources to sort through, making this method impractical. We also excluded studies that included data collected at CRP sites but did not evaluate CRP *per se* (e.g., using previously collected CRP data to test the efficacy of a tool or model without drawing inferences about outcomes associated with CRP).

The literature search retrieved 48 review papers examining the effect of CRP but putting forward no novel data. These review papers, which ranged from systematic meta-analyses to literature reviews specific to a particular topic, were not included in our study counts as the data they contained would be captured in other studies collected in our database. We could not access a small number of papers retrieved in our search (n = 43), and information from the title, abstract, and keywords of these papers was not sufficient to include them in our final database. These studies were either published in smaller, trade specific publications that we were not able to access or were published prior to 2000 making it possible that they do not exist in a viewable form online.

Study categorization

After filtering out unsuitable papers, the remaining studies (n = 577; Table S1) were broadly categorized into 5 major groups: Vegetation, Wildlife, Air/Soil/Water, Social and Productivity. The first three groups include studies measuring the effect of CRP on non-crop plants, animals, and air, soil and/or water respectively. We chose to combine papers examining air, soil and water as many of the papers we collected measured similar responses (e.g., carbon

content, nutrient content) in more than one of these categories, and in some cases, responses included aspects of more than one of these components (e.g., sediment runoff involves both soil and water). The Productivity category included studies examining land use or agricultural productivity, the latter of which could be measured in either dollars or crop biomass. The Social category included papers studying the behavioral responses associated with CRP contracts (e.g., producer choices in CRP auctions), and other individual and community effects of CRP. These five categories were not mutually exclusive, and one study could be eligible for multiple categorizations depending on its scope. For consistency and to avoid editorializing the findings that were evaluated, we based categorizations on the primary results presented in the study. For example, plant height can be both a measure of wildlife habitat and plant physiology. If a study presented results as “plant height,” we would consider it to be a vegetation study, whereas if the same measure was presented as habitat structure or habitat quality, we would consider it to be a wildlife study.

For each eligible study, we extracted the following data if available: location data, including study site, and state, the duration of the study and season when data were collected, and CRP-specific information such as the number of CRP sites studied, CP practice (e.g., CP1, CP2, etc.), and the number of years sites were enrolled in the program. We compared the final number of studies conducted in each state to total CRP acreage (USDA-FSA 2017) to determine if more CRP studies have been conducted in states with larger shares of CRP land. For Vegetation and Wildlife studies, we also noted the types of reported results, which we grouped into general categories for further classification (Table 1).

Table 1: Vegetation and Wildlife results reported by studies.

**Vegetation**		
**Name**	**Description**	**Examples**
Biomass	Measure of plant or plant tissue to establish the amount in a given space	Biomass, Abundance
Cover	Measurement of space on land taken up by plant or plant type	Percent cover, percent grass
Diversity	Measure of number or composition of plant species	Diversity, Evenness, Species composition
Disease	Measure of disease severity or plant response to disease severity	Number of spots, pathogen cell counts
Physiology	Measure of plant traits at individual plant level from cellular to whole plant	Growth rate, Number of leaves
Reproduction	Measure of seed/fruit production and biomass	Number of seeds, seed size, fruit size
Structure	Measure of height taken up by vegetation	VOR, vegetation height
**Wildlife**		
**Name**	**Description**	**Examples**
Abundance	Measure of number or biomass of wildlife	Biomass, Abundance
Diet	Measure of amount or richness of food being utilized by wildlife	Food diversity, Foraging Rates
Distribution	Measure of density, movement or occurrence of wildlife	Habitat Use, Detection Probability, Occupancy
Diversity	Measure of number or composition of wildlife species	Diversity, Evenness, Species composition
Ecological Service	Measure of specific ecosystem services provided by the wildlife being studied	Avian conservation value, Honey Yield, Pollination
Mortality	Measure of mortality or predation rate of wildlife	Mortality, Predator rates, Survival
Reproduction	Measure of number and survival of wildlife broods	Brood Count, Fecundity, Nest Success

Given the threat to biodiversity from land cover change noted earlier, we also collected more detailed information about CRP studies categorized as either vegetation or wildlife. Specifically, we noted if particular species were reported in the results. We used scientific names for each organism if provided. If only common names were provided, then the scientific name was retrieved and used. All names were checked and updated to present-day nomenclature. We also recorded whether vegetation studies made comparisons that we considered to be especially relevant ecologically, including comparisons of warm season (C4) vs. cool season (C3) grasses, and native vs. non-native plants (all specific comparisons in Table 2). For these vegetation comparisons, we were only interested in CRP treatments, so non-CRP plots included in studies were not included in these comparisons.

Table 2: Types and number of comparisons made in vegetation studies.

Conservation Practices	# Studies	Functional Groups	# Studies	Management	# Studies
CRP Age	20	Cool-warm/C3-C4 grasses	8	Grazed	9
CRP Type	12	Native-Non-Native	3	Herbicide	9
		C4-other	1	Hayed	7
		Interseeding	1	Mowed	6
		Old World bluestem-native mixed species	1	Burned	5
		Seral-Native	1	Disc	5
				Fertilizer	3
				Harvested	3
				Previous crops	1
				Tillage	1
				Wooded	1

The majority of wildlife studies in our database focused on birds. To assess conservation status of bird species that have been studied in the context of CRP, we utilized the Avian Conservation Assessment Database (ACAD) (Partners in Flight 2024). These scores designate species of greatest conservation concern due to small and/or rapidly declining populations, limited distributions, and high threats throughout their ranges. Species in the Red Watchlist are of highest concern, followed by Orange then Yellow. A full explanation for how each color coding is scored is available on the ACAD website (Partners in Flight 2024).

Results

Spatial and temporal trends in CRP research

Of the 577 studies collected in our database the largest group was Air/Soil/Water (188) followed by Wildlife (169), Productivity (162), Vegetation (77) and Social (56) (Figure 1). The earliest study captured in our search that contained the phrase “Conservation Reserve Program” was published in 1985, but it did not include an examination of CRP effects as the program was

established that year. The first studies explicitly evaluating CRP effects are from 1987, including studies modeling agricultural productivity and program payouts, as well as studies examining CRP effects on air, soil and water (Figure 2). In 1990 the first studies examining the effect of CRP on farmer decisions as well as public opinion of CRP (Social) were published (Figure 2). Studies examining effects of CRP on vegetation and wildlife began to be published in 1993 (Figure 2). During the 30-year period between 1994 and 2023 an average of 20 studies were published per year ranging from 11 studies in 1995 to 36 studies in 2016.

Figure 1: Number of studies from each category collected in our database. Some studies are counted more than once in this graph because they fall under multiple study categories.

Figure 2: Number of studies from each category collected in our database separated by publication year. Some studies are counted more than once in this graph because they fall under multiple study categories. Studies from 2024 are not well represented as our literature search began in early February 2024.

Regarding the spatial distribution of CRP research, Kansas has had the most published studies overall (Figure 3) as well as the most vegetation and wildlife studies (Figure 4). Kansas and North Dakota both had the most Productivity studies (Figure 4). Illinois has hosted the most Social studies of any state, while Nebraska has had the most studies on Air, Soil and Water (Figure 4). The majority of studies have occurred near the 98th meridian, with most of the studies in our dataset occurring in Great Plains states (Figure 3). When comparing this spatial distribution of studies to CRP enrollment (Table S2), states with high CRP acreage tend to also have high numbers of CRP studies, with the top 10 states in terms of enrollment averaging 54

studies each; 64% (n = 320) of all studies that included a specified location occurred at least in part in these 10 states. The most obvious exception to this pattern is Washington, which had fewer CRP studies than expected based on enrollment; this state ranked seventh in terms of CRP acreage but 16th in number of studies with 26 total. Conversely, Wisconsin and Michigan had more studies than expected based on their share of enrollment, ranking 24th and 30th respectively in terms of CRP acreage but 14th and 13th in number of studies (Table S2). Our database contained 82 studies that were described as using “National” level data, meaning the CRP data in the study was likely collected from all or most states with land enrolled in CRP. However, the specific states represented in the data were not specified in these national studies, preventing inclusion in our state counts. Most national studies (50 studies; 62%) were in the Productivity category, typically utilizing nationwide surveys or satellite images of land cover, 21 studies (26%) were in the Air/Soil/Water, 13 were categorized as Social (16%), and 8 (10%) were categorized as Wildlife with no national scale Vegetation studies. We did not find any studies pertaining to CRP for three states (Arizona, Nevada and Hawaii) or for Puerto Rico, which also participates in the program (Figure 3).

Figure 3: Map depicting total number of studies of CRP effects that occurred in each state. This does not include 85 studies that included “National” level data, which did not specify what state or states the data was collected in.

Figure 4: Map depicting number of studies that occurred in each state, separated by study category. This does not include 85 studies that included “National” level data, which did not specify what state or states the data was collected in.

Vegetation and wildlife responses to CRP

We found 77 studies that examined vegetation including measurements of grasses, forbs, trees, shrubs, and mixed communities capturing more than one of the previous plant types. However, only 14 of these studies specified the plant group evaluated (e.g., grass, tree) and only 9 were explicit about the species or genus of interest (e.g., *Asclepias spp.*, *Panicum virgatum*, *Pinus palustris*), so we were unable to further examine trends in types of vegetation studied. Finally, we retrieved 169 studies that measured CRP effects on wildlife (e.g., effects on abundance, behavior, or reproduction) or wildlife habitat (e.g., habitat use or quality). All wildlife studies were explicit about the organism(s) of interest (i.e., bird, fish, invertebrate, mammal or reptile), and wildlife research covered a wide range of topics including population dynamics, behavior, and ecosystem services pertaining to the species of interest (e.g., pollination, pest removal).

Of all studies in these groups, we found 75 vegetation studies (97%) and 151 wildlife studies (89%) that reported study duration. Vegetation studies ranged from 1 to 9 years in duration (average = 2 years, median = 2 years), while wildlife studies ranged from 0.5 to 23 years (average = 2.5 years, median = 2 years). For both groups, the most common study duration was one year (30 of 75 vegetation and 20 of 151 wildlife studies).

Among 77 studies that included descriptions of the specific vegetation variables assessed in the context of CRP, most (n = 54) included vegetation cover measurements (Figure 5). Most studies (n=68) did not explicitly examine CRP effects on a particular species, but the few that did tended to evaluate either invasive species (e.g., *Ventenata dubia*), species of conservation concern (e.g., *Pinus palustris*), or species useful for agriculture (e.g., *Panicum virgatum*).

Regarding vegetation comparisons, 23 studies (29%) did not include vegetation comparisons in our previously identified categories deemed ecologically important (Table 2). Studies that did include such vegetation comparisons fell into 3 broad categories, including comparing outcomes for: fields enrolled in different periods or different CRP implementations (e.g., comparing number of years in CRP program or under specific CP practices); plants in different functional groups (e.g., C3 to C4 grasses, native to non-native species); or different management approaches (e.g., hayed, disced, burned) (Table 2).

Figure 5: Number of Vegetation studies from each category collected in our database. Some studies are counted more than once in this graph because they fall under multiple study categories.

The majority of wildlife studies (n= 107) evaluated how CRP was associated with some measure of animal abundance (e.g., average counts, biomass; Table 1, Figure 6). The next most common measures were reproduction (n = 42), diversity (n = 37), and distribution (n = 27) (Table 1, Figure 6). We found very few studies focused on wildlife ecosystem services (n = 9), mortality (n = 6), or diet (n = 5). Nearly all wildlife studies pertained to birds (n = 115; Figure 7). The next most-frequently studied taxa were invertebrates (n = 36); most invertebrate studies focused on insects, but some evaluated other terrestrial invertebrates such as arachnids and annelids (Figure 7). We also collected 18 studies on mammals, which tended to focus on deer, coyote, and rodents like prairie dogs and prairie mice (Figure 7). We only found a single study focused on fish or reptiles (Figure 7) and did not find any studies examining other important wildlife groups such as amphibians as well as important mammal groups like bats.

Figure 6: Number of Wildlife studies from each category collected in our database. Some studies are counted more than once in this graph because they fall under multiple study categories.

Figure 7: Number of Wildlife studies examining major animal groups. Some studies are counted more than once in this graph because they examined more than one type of animal.

Among the 115 studies examining birds, 160 unique species were studied (Table S3); of these, 55 species were only reported in a single study. The majority of the bird species studied were in the order Passeriformes (songbirds; 104 species) followed by Charadriiformes (shorebirds like sandpipers and plovers; 11 species), Galliformes (e.g., turkey, pheasant, and grouse; 10 species) and Anseriformes (waterfowl; 9 species). Grasshopper Sparrow (*Ammodramus savannarum*) was the most studied species (50 studies), followed by Dickcissel (*Spiza americana*; 41 studies) and Ring-necked Pheasant (*Phasianus colchicus*; 38 studies) (Table 3). When considering ACAD's watchlist, only 25 species (16% of those studied) were on the list, including 9 species on the Red Watch List, 4 on the Orange Watch List and 12 on the Yellow Watch List (Table S3). The three most frequently studied species with a watch list designation were Bobolink (*Dolichonyx oryzivorus*; Orange Watch List; 28 studies), Field Sparrow (*Spizella pusilla*; Yellow Watch List; 20 studies), and Henslow's Sparrow (*Centronyx henslowii*; Yellow Watch List; 15 studies). Lesser Prairie-Chicken (*Tympanuchus pallidicinctus*; 12 studies) was the most commonly studied Red Watch List species. The 25 species on ACAD's watchlist appeared in 5 studies each on average (range 1 to 28) with 9 of the species appearing in only a single study (Table S3).

Table 3: Names and taxonomic information of the 10 most commonly studied birds.

Species	Common Name	Family	Order	# Studies
Ammodramus savannarum	Grasshopper Sparrow	Passerellidae	Passeriformes	50
Spiza americana	Dickcissel	Cardinalidae	Passeriformes	41
Phasianus colchicus	Ring-Necked Pheasant	Phasianidae	Galliformes	38
Agelaius phoeniceus	Red-Winged Blackbird	Icteridae	Passeriformes	35
Molothrus ater	Brown-Headed Cowbird	Icteridae	Passeriformes	35
Zenaida macroura	Mourning Dove	Columbidae	Columbiformes	31
Sturnella magna	Eastern Meadowlark	Icteridae	Passeriformes	31
Geothlypis trichas	Common Yellowthroat	Parulidae	Passeriformes	30
Dolichonyx oryzivorus	Bobolink	Icteridae	Passeriformes	28
Sturnella neglecta	Western Meadowlark	Icteridae	Passeriformes	27

Discussion

We conducted the first scoping review of peer-reviewed journal publications of CRP research with the goal of quantifying trends in the literature and identifying potential gaps in the research on this important and long-standing private lands conservation program. Our analysis uncovered several trends: 1) The published research on CRP has been relatively consistent temporally since the program's inception in 1985, and the majority of research has been conducted in regions that have had the greatest share of CRP enrollment (the Great Plains and upper Midwest), 2) The largest share of the research has pertained to CRP's effect on air, soil and water, and vegetation studies are the least common, 3) The focus of vegetation and wildlife studies has largely been on vegetation cover and wildlife abundance and the vast majority of

wildlife-related CRP studies have evaluated bird responses to the program, with a marked lack of studies on fish, reptiles, and amphibians.

Spatial and Temporal Trends in CRP Research

We did not see any major year-over-year changes in the type or number of studies published about CRP since the program's inception in 1985. Beginning in 1993 we observed a relatively consistent number of studies in all 4 categories. Although CRP enrollment has declined by more than 10 million acres since 2007 (USDA-FSA 2020), we did not observe a concomitant decline in published CRP research. These consistent rates of publication concurrent to declining CRP enrollment may reflect trends in wildlife-focused research, and specifically, an increase of this research on existing CRP-enrolled land toward taxa that were previously understudied, both on CRP lands and more broadly. For example, prior to 2013 only a single paper published in our database (Davros *et al.* 2006) focused on insects outside the context of plant pests or vertebrate prey. Starting in 2013 these insect papers began to capture a much wider gamut of topics including insect community structure, biodiversity, and pollinator nutrition; with only 3 papers from this time period primarily covering insects as prey or pests. Perhaps the larger number of studies on the effects of CRP on insects reflects recently shifting priorities concerning the importance of invertebrates, especially of the ecosystem functions and services provided by pollinators (Allen-Wardell *et al.* 1998; Vanbergen *et al.* 2013).

Our scoping review uncovered clear spatial patterns in CRP studies, with most conducted in states on or near the 98th meridian (Figure 3,4). These states also tend to have the most CRP land; specifically, the 6 most-frequently studied states (North Dakota, South Dakota, Nebraska, Kansas, Oklahoma and Texas) are in the top 15 in terms of CRP land acreage (Table S2), and

surrounding states like Iowa, Montana and Minnesota also have relatively high CRP acreage and numbers of studies (Table S2). However, not all states followed this trend. For example, some states in the Great Lakes region (Wisconsin and Michigan) had a high number of studies relative to their CRP enrollment, and Pacific Northwest States (Washington and Oregon) had relatively few studies relative to enrollment. Identifying the factors driving these regional patterns is beyond the scope of this study, but these spatial trends may relate to applicability of certain conservation practices to certain regions (e.g., cropland buffers are less relevant in areas with less land devoted to crops)—and thus the availability of funding to study program effects in areas highly important to the program—as well as to the population of researchers available to study CRP in universities and other research institutions within each state. Despite the wealth of research presented herein, underrepresentation in some states may be problematic from a management perspective. Species and ecosystems respond not only to land management but also to interactions between land management and underlying topoedaphic and climatic regimes (Burruss et al., 2023; Duchardt et al., 2018; Porensky & Blumenthal, 2016). For example, the Grasshopper Sparrow, which we found to be the most frequently studied bird in the context of CRP, has been shown to respond positively to disturbances like fire and grazing at the eastern edge of its range, but to respond negatively to these disturbances at the western edge of its range (Shaffer *et al.* 2021). The Pacific Northwest and other understudied regions differ ecologically and culturally from regions where most CRP research has occurred, and the nature of CRP benefits are similarly expected to be regionally variable. As such, additional research may be necessary in underrepresented regions or for underrepresented outcomes, especially as CRP enrollment and practices change over time.

CRP studies of Air/Soil/Water, Productivity, and Social Aspects

It is perhaps not surprising that one of the largest categories of CRP research was "Productivity", as we defined many broad and important study subjects to fall under this category (e.g., agricultural productivity, land use, economic output,). While CRP is a land restoration program, studies are needed that examine changes in its adoption by agricultural producers, as well as behavioral responses of producers to changes in the program. Additionally, we found 56 studies using surveys and interviews to understand how stakeholders respond to CRP as well as their opinion of the program. Quantifying the value of CRP to individuals and communities often involves surveys to elicit information on the value of the environmental benefits it provides. Measuring and articulating these benefits is necessary for adaptive management within the program, and for improving outcomes for farmers, local communities, and the environment (Everard 2016; Kelsey *et al.* 2018; Saturday 2018).

Major goals of the CRP program are reducing soil erosion and improving air and water quality. We found that the largest group of studies (35%) in our database examined the effect of CRP on a combination of air, soil, and water (Figure 1). Since the program's inception many studies have confirmed that CRP adoption reduces air pollution and greenhouse gas emissions (Abraha *et al.* 2018; Becker *et al.* 2022; Ruan & Philip Robertson 2013), soil erosion (Hoekman *et al.* 2018) and water degradation (Yin *et al.* 2021), and also provides flood risk mitigation (Jobe *et al.* 2018) and increased carbon retention (Johnson *et al.* 2005). Carbon in particular seemed to be a major focus of these studies, with papers examining how carbon retention in CRP-enrolled lands is affected by soil and water use (Amoakwah *et al.* 2022; Sainju *et al.* 2010; Yang *et al.* 2022), land use (Obembe & Hendricks 2022), and productivity (Abraha *et al.* 2019; Zenone *et al.* 2013).

A broader synthesis on how soil, water and other aspects of habitat quality were improved is needed. Which soil parameters were improved (N? P? pH?) ? Which air quality measures were improved? The social aspects should be separated out and a synthesis needs to be provided on broadly which social aspects were improved.

Vegetation and Wildlife Responses to CRP

Restoring wildlife habitat is also an important CRP goal, and studies of vegetation and wildlife outcomes were well represented in our database, with 77 vegetation and 169 wildlife studies. Vegetation studies were found to be the least represented of the 4 broad categories. This may be because fostering vegetation (e.g., diversity, cover) per se is not a major goal of CRP, and instead is a means to achieve outcomes that are captured in our other study categories (e.g., reduction of soil erosion, enhancement of wildlife habitat). However, in recent years, increasing understanding of the role of plant functional groups, or even of specific plant species, in providing such ecosystem services, is leading to a greater interest in vegetation-focused outcomes. The current iteration of CRP now includes several conservation practices that encompass land restoration activities in multiple vegetation and land cover types including wetlands, riparian areas, forests and shrublands. Although studies of effects of CRP on these cover types exist among the papers in our dataset, most research still appears to focus primarily on grasslands, and to compare CRP to other land uses, especially agricultural uses. Research evaluating how a broad range of ecosystem types respond to surrounding CRP lands would provide insight into how these CRP vegetation communities are responding to ongoing anthropogenic changes such as nutrient enrichment or climate change.

Wildlife in particular have become a larger priority in the CRP program over time (https://www.nrcs.usda.gov/ceap/wildlife), as reflected both by trends in the literature, and by establishment of newer CRP practices, starting in the mid-2000s, that allow stakeholders to directly curate lands to benefit ecologically important wildlife species (CP38) including beneficial birds (CP33, CP37) and pollinators (CP42) (USDA-FSA 2024). CRP supports wildlife

through increasing habitat area and quality (Rey Benayas & Bullock 2012, 2015), which in turn generates a number of ecosystem services for humans. For example, the third most-commonly studied bird species in our dataset was a game species (ring-necked pheasant), and other game species included greater prairie-chicken, northern bobwhite, white-tailed deer, wild turkey, and several duck species. Game species provide meat and sport for millions of stakeholders and, through hunting licenses, provide funding for wildlife conservation through the Pittman-Robertson Act (Crafton 2019). Game and non-game species alike also provide numerous ecosystem services that benefit the environment and society, including pest removal, pollination, and wildlife viewing (Otto et al., 2020; Newton et al., 2021; Walston et al., 2024). Our review illustrates that research has addressed effects of CRP on a wide variety of game and non-game wildlife species; however, we found few wildlife and vegetation studies that directly measured ecosystem services, and those that did mainly examined insect pollinators (see further discussion of pollinators below). Further research that evaluates the wildlife and vegetation-mediated ecosystem services provided by CRP could further increase understanding of the role of CRP in providing benefits to the environment and humans.

I would have wanted more discussion/synthesis on the effects of the CRP on wildlife. Specific examples such as "species A increased due the program in the State X" or "community diversity or overall biomass of a species increased in riparian habitats"... this would make the discussion more informative compared to whether 'n' number of studies addressed issues relating to biomass and diversity.

The vegetation and wildlife research in our database was composed mostly of short-term studies, with vegetation studies averaging 2 years in duration, wildlife studies averaging 2.5 years, and with the largest number of studies in both categories focused on a single year. This short-term study duration is expected as funding allotments and job appointments (e.g., graduate student positions) tend to only cover increments of a few years. However, both long-term and large-scale studies are important for accurately measuring ecological trends and capturing lag effects that might not manifest until years after management is applied (Wauchope *et al.* 2021; White 2019). Further, long-term studies, despite being rarer, are disproportionately cited in the

Ok. But what do some of these short term studies driven by graduate students demonstrate. Some synthesis would be essential

literature and by policy makers (Hughes *et al.* 2017). Despite the challenges inherent in long-term research (Kuebbing *et al.* 2018), such efforts are warranted in the context of CRP. Ecological restoration, which is a key desired outcome of CRP from a wildlife habitat perspective, is an inherently dynamic process (Hilderbrand *et al.* 2005; Hobbs & Harris 2001). Assumptions about predictability of land restoration (Carbon Copy Myth; Hilderbrand et al. 2005) or immediate responses of wildlife to newly-restored habitat (Field of Dreams Myth; Hilderbrand et al. 2005) may not be met, and it may take time to observe these responses in vegetation and wildlife. Further, the longer-term ecological studies that have been conducted sometimes reveal different, or even opposing outcomes relative to shorter-term studies (e.g., Dohm et al., 2019). Thus, care should be taken in interpreting short-term snapshot studies, and CRP studies over a longer duration could examine the dynamic effects of CRP (e.g., evaluate outcomes relative to time since CRP enrollment).

We noted several additional gaps and emphases in wildlife-focused CRP studies that may also help to inform future research. The majority of Wildlife studies focused on measures of wildlife abundance like density, abundance, or biomass of all individuals (Figure 6). Metrics related to abundance and density are the main currency of conservation and population management activities, and also the center of many methodological discussions (Callaghan *et al.* 2024; Nichols & MacKenzie 2004), but using abundance alone to inform management decisions can in some cases be misleading. For example, if animals are drawn to habitats that decrease survival (Battin 2004), abundance may be a confounding indicator of habitat quality and success of land management activities, including those associated with CRP. Further, underlying drivers of behavior (Wilson *et al.* 2020) or habitat quality (Johnson 2007) may obscure interpretations of abundance trends. Researchers should balance the focus on local abundance with integration of

So, make this more informative by picking some examples of species that were increased due to either land management acitivies but was mis-interpretted as a those associated with CRP.

outcomes that more directly capture population trends and indicators of community health (i.e., reproductive output). Alternatively, a more holistic understanding of large-scale abundance trends in response to CRP and other land management approaches would ensure that abundance metrics more adequately capture overall population status in response to management.

We also found biases in the types of wildlife studied in CRP publications, with birds being included in the majority of studies (68%) (Figure 7). This emphasis on birds has been shown to exist across the ecological literature (Troudet *et al.* 2017), and likely reflects societal and funding agency interests, ease of monitoring, as well as the diverse ecosystem services provided by birds, including pest control, seed dispersal, pollination, and aesthetic value (Whelan *et al.* 2008). However, there is clearly a need to evaluate CRP effects on other, lesser-studied organisms. Amphibians in particular were not studied in any of the publications retrieved in our study, although we are aware of at least one amphibian study not retrieved using our formal search terms (Reeves *et al.* 2016). Amphibians have declined in recent years for a number of reasons including habitat degradation associated with agricultural intensification (Swartz & Miller 2019; Youngquist & Boone 2014). Additional research focusing on understudied wildlife groups can provide a more complete understanding of how CRP is affecting wildlife communities as a whole.

I agree that more emphasis needs to be placed on other taxa. However, what do some of the 68% of biased studies show? Are some of the non-native species primarily benefitted or are some of the common species increased? Please provide specific examples and sythesize the information about overall conclusions of trends made by the authors of the study.

North American birds, including many species of grassland-dependent birds, are declining at alarming rates (Rosenberg *et al.* 2019), and this may be an additional reason for the focus of CRP research on birds. Of the 160 bird species observed in our database, 135 (85%) are not listed under any of the ACAD watchlist designations (i.e., the Red, Orange, or Yellow Watchlists). Of the 10 most-frequently studied bird species, only Bobolink (Dolichonyx oryzivorus) had a watchlist designation. Of the remaining Watchlist birds that have been studied,

For example? which non-listed species of the ACAD watch list were considered?

only 6 had been studied in more than 5 instances: Field Sparrow (Spizella pusilla), Henslow's Sparrow (Centronyx henslowii), Lesser Prairie-Chicken (Tympanuchus pallidicinctus), Chestnut-collared Longspur (Calcarius ornatus), Baird's sparrow (Centronyx bairdii), Greater prairie-chicken (Tympanuchus cupido). Many of these species are believed to have benefitted substantially from CRP, with steep declines through much of the 20th century appearing to have stabilized to some degree in part due to efforts to restore their habitat (Herkert 2007; Tanner *et al.* 2021). This high number of avian studies includes many examples of species benefiting from CRP land. Additional research that includes other species, both avian as well as non-avian wildlife, can help to bolster conservation efforts and add to the list of CRP success stories.

OK

What is the overall nature of the benefit to these species: did their densities increase? Did they have higher reproductive success?

Study limitations

While all of the studies in our database examined the effect of CRP, many did not include information useful to addressing our objectives of characterizing trends in the literature. For example, of the 577 studies only 9% (n = 56) reported the specific CRP conservation practices that were carried out on the enrolled CRP lands in the study. As previously mentioned, early CRP practices primarily consisted of planting prescribed grass mixes (CP1, CP2); therefore, authors of early studies may have felt it was unnecessary to report specific Conservation Practices. It is also possible that details about the CRP enrollments studied were confidential and thus not available to researchers even when they had permission to conduct research on CRP lands. Other details like age, size and number of CRP sites were infrequently reported, or were averaged across multiple sites, making it difficult to extract this information precisely. Because the CRP is not one-size-fits-all, reporting of CRP CP practices in future studies is vital both for

researchers attempting to measure CRP benefits and shareholders seeking to understand best practices relative to their land-use goals.

Our database only included studies in which the term "Conservation Reserve Program" was used in a searchable way (i.e., in the title, abstract, or keywords). We also note that our database only includes studies that explicitly evaluated effects of CRP directly. Many other studies that we excluded used CRP land but either did not specifically evaluate effects of CRP or conflated the program with other similar land use practices, making it impossible to isolate unique effects of CRP. We also excluded studies not published in peer-reviewed journal articles (e.g., theses, government reports), which may represent a substantial fraction of CRP studies. Although such publications can contain information that is highly useful for conservation and policy activities, this gray literature is not indexed and searchable in the same way as databases of journal articles (e.g., Web of Science), and is therefore more difficult and time consuming to thoroughly capture. Notwithstanding these limitations in data availability, formal meta-analyses of CRP effects and leveraging the wealth and diversity of CRP research that we have highlighted in this scoping review, would provide broader scale understanding of CRP's benefits as well as the moderating factors influencing its benefits. Factors that could be evaluated using meta-analyses include, for example, those related to regional variation, conservation practices used, methods of research employed, and specific endpoints evaluated (e.g., for wildlife and vegetation, the taxonomic groups studied).

Conclusion

This scoping review of the Conservation Reserve Program (CRP) literature highlights the significant contributions of CRP to environmental conservation, agricultural productivity, and

socioeconomic benefits. The research spans multiple dimensions, including wildlife habitat restoration, environmental quality improvement, and carbon sequestration. The majority of studies focused on air, soil, and water, while vegetation studies and social surveys were the least represented. Notably, most wildlife research has focused on birds, leaving a gap in studies on fish, reptiles, amphibians, and other less-studied taxa. The review also reveals a geographic concentration of research in the Great Plains, underscoring the need for more studies in other ecologically distinct and important regions like the Pacific Northwest. Addressing these gaps, particularly with more long-term studies and a broader range of taxa and regions, will be essential to fully understand and enhance the CRP's potential to support biodiversity and ecosystem services across the U.S.

Acknowledgments

This research was funded by the U.S. Department of Agriculture (USDA) Natural Resources Conservation Service (Award # NR233A750023C009), the USDA Economic Research Service (Agreement #58-6000-2-0071), and USDA Hatch Grant funds through the Oklahoma Agricultural Experiment Station (OKL03231 and OKL02915). We thank all authors whose work was included in our scoping review. The findings and conclusions in this presentation are those of the author(s) and should not be construed to represent any official USDA or U.S. Government determination or policy.

Statement of authorship: All authors contributed to developing the idea for this paper; M.P.N. led the database construction and curation, analyses, and visualization with input from C.J.D and S.R.L, and writing the first draft; K.M. and R.I. provided recommendations for the late drafts of

this manuscript, and C.J.D and S.R.L developed the idea for this paper, visualization, writing, review, and editing. All authors contributed to and approved the final version.

Citations

Abraha, M., Gelfand, I., Hamilton, S.K., Chen, J. & Robertson, G.P. (2019). Carbon debt of field-scale conservation reserve program grasslands converted to annual and perennial bioenergy crops. *Environmental Research Letters*, 14.

Abraha, M., Hamilton, S.K., Chen, J. & Robertson, G.P. (2018). Ecosystem carbon exchange on conversion of Conservation Reserve Program grasslands to annual and perennial cropping systems. *Agric For Meteorol*, 253–254, 151–160.

Allen-Wardell, G., Bernhardt, P., Bitner, R., Burquez, A., Buchmann, S., Cane, J., *et al.* (1998). *The Potential Consequences of Pollinator Declines on the Conservation of Biodiversity and Stability of Food Crop Yields*. *Biology (Basel)*.

Amoakwah, E., Lucas, S.T., Didenko, N.A., Rahman, M.A. & Islam, K.R. (2022). Impact of deforestation and temporal landuse change on soil organic carbon storage, quality, and lability. *PLoS One*, 17.

Battin, J. (2004). When good animals love bad habitats: Ecological traps and the conservation of animal populations. *Conservation Biology*.

Baylis, K., Coppess, J., Gramig, B.M. & Sachdeva, P. (2022). Agri-environmental Programs in the United States and Canada. *Rev Environ Econ Policy*, 16, 83–104.

Becker, D.A., Maas, A., Bayham, J. & Crooks, J. (2022). The Unintended Benefits of the Conservation Reserve Program for Air Quality. *Geohealth*, 6.

Begosh, A., Smith, L.M. & McMurry, S.T. (2022). Major land use and vegetation influences on potential pollinator communities in the High Plains of Texas. *J Insect Conserv*, 26, 231–241.

Bryan, B.A., Gao, L., Ye, Y., Sun, X., Connor, J.D., Crossman, N.D., *et al.* (2018). China’s response to a national land-system sustainability emergency. *Nature*, 559, 193–204.

Burruss, N.D., Peters, D.P.C. & Huang, H. (2023). The resistance and resilience of Great Plains ecoregion boundaries to the 1930s drought as a lens to future dynamics. *Ecosphere*, 14.

Callaghan, C.T., Santini, L., Spake, R. & Bowler, D.E. (2024). Population abundance estimates in conservation and biodiversity research. *Trends Ecol Evol*.

Caro, T., Rowe, Z., Berger, J., Wholey, P. & Dobson, A. (2022). An inconvenient misconception: Climate change is not the principal driver of biodiversity loss. *Conserv Lett*, 15.

Chazdon, R.L., Lindenmayer, D., Guariguata, M.R., Crouzeilles, R., Benayas, J.M.R. & Chavero, E.L. (2020). Fostering natural forest regeneration on former agricultural land through economic and policy interventions. *Environmental Research Letters*, 15, 43002.

Crafton, R.E. (2019). Pittman-Robertson Wildlife Restoration Act: understanding apportionments for states and territories. *Congressional Research Service*, 45667, 1–34.

Davros, N.M., Debinski, D.M., Reeder, K.F. & Hohman, W.L. (2006). Butterflies and Continuous Conservation Reserve Program Filter Strips: Landscape Considerations. *Wildl Soc Bull*, 34, 936–943.

Dohm, R., Jennelle, C.S., Garvin, J.C. & Drake, D. (2019). A long-term assessment of raptor displacement at a wind farm. *Front Ecol Environ*, 17, 433–438.

Duchardt, C.J., Porensky, L.M., Augustine, D.J. & Beck, J.L. (2018). Disturbance shapes avian communities on a grassland–sagebrush ecotone. *Ecosphere*, 9.

Everard, M. (2016). Conservation Reserve Program (CRP): Example of Land Retirement. In: *The Wetland Book*. Springer Netherlands, pp. 1–4.

FAO. (2024). *FAOSTAT Database*. *https://www.fao.org/faostat/en/#data*.

Fayet, C.M.J., Reilly, K.H., Van Ham, C. & Verburg, P.H. (2022). The potential of European abandoned agricultural lands to contribute to the Green Deal objectives: Policy perspectives. *Environ Sci Policy*, 133, 44–53.

Gelfand, I., Zenone, T., Jasrotia, P., Chen, J., Hamilton, S.K. & Robertson, G.P. (2011). Carbon debt of Conservation Reserve Program (CRP) grasslands converted to bioenergy production. *Proc Natl Acad Sci U S A*, 108, 13864–13869.

Herkert, J.R. (2007). Conservation Reserve Program Benefits on Henslow's Sparrows Within the United States. *J Wildl Manage*, 71, 2749–2751.

Hilderbrand, R.H., Watts, A.C. & Randle, A.M. (2005). The Myths of Restoration Ecology. *Ecology and Society*, 10.

Hobbs, R.J. & Harris, J.A. (2001). Restoration Ecology: Repairing the Earth's Ecosystems in the New Millennium. *Restor Ecol*, 9, 239–246.

Hodur, N.M., Leistritz, F.L. & Bangsund, D.A. (2002). Local Socioeconomic Impacts Of The Conservation Reserve Program. *Agribusiness & Applied Economics Report* , 476.

Hoekman, S.K., Broch, A. & Liu, X. (Vivian). (2018). Environmental implications of higher ethanol production and use in the U.S.: A literature review. Part I – Impacts on water, soil, and air quality. *Renewable and Sustainable Energy Reviews*.

Hughes, B.B., Beas-Luna, R., Barner, A.K., Brewitt, K., Brumbaugh, D.R., Cerny-Chipman, E.B., *et al.* (2017). Long-Term studies contribute disproportionately to ecology and policy. *Bioscience*.

Jaureguiberry, P., Titeux, N., Wiemers, M., Bowler, D.E., Coscieme, L., Golden, A.S., *et al.* (2022). *The direct drivers of recent global anthropogenic biodiversity loss*. *Sci. Adv*.

Jobe, A., Kalra, A. & Ibendahl, E. (2018). Conservation Reserve Program effects on floodplain land cover management. *J Environ Manage*, 214, 305–314.

Johnson, J.M.F., Reicosky, D.C., Allmaras, R.R., Sauer, T.J., Venterea, R.T. & Dell, C.J. (2005). Greenhouse gas contributions and mitigation potential of agriculture in the central USA. In: *Soil and Tillage Research*. Elsevier, pp. 73–94.

Johnson, K.A., Dalzell, B.J., Donahue, M., Gourevitch, J., Johnson, D.L., Karlovits, G.S., *et al.* (2016). Conservation Reserve Program (CRP) lands provide ecosystem service benefits that exceed land rental payment costs. *Ecosyst Serv*, 18, 175–185.

Johnson, M.D. (2007). Measuring habitat quality: A review. *Condor*.

Kelsey, R., Hart, A., Scott Butterfield, H. & Vink, D. (2018). Groundwater sustainability in the San Joaquin Valley: Multiple benefits if agricultural lands are retired and restored strategically. *Calif Agric (Berkeley)*, 72, 151–154.

Kong, L., Wu, T., Xiao, Y., Xu, W., Zhang, X., Daily, G.C., *et al.* (2023). Natural capital investments in China undermined by reclamation for cropland. *Nat Ecol Evol*, 7, 1771–1777.

Kuebbing, S.E., Reimer, A.P., Rosenthal, S.A., Feinberg, G., Leiserowitz, A., Lau, J.A., *et al.* (2018). Long-term research in ecology and evolution: a survey of challenges and opportunities. *Ecol Monogr*, 88, 245–258.

Lizotte, R.E., Yasarer, L.M.W., Locke, M.A., Bingner, R.L. & Knight, S.S. (2017). Lake Nutrient Responses to Integrated Conservation Practices in an Agricultural Watershed. *J Environ Qual*, 46, 330–338.

Locke, M.A., Lizotte, R.E., Yasarer, L.M.W., Bingner, R.L. & Moore, M.T. (2020). Surface runoff in Beasley Lake watershed: Effect of land management practices in a Lower Mississippi River Basin watershed. *J Soil Water Conserv*, 75, 278–290.

Moore, J.M., Manter, D.K., Bowman, M., Hunter, M., Bruner, E. & McClelland, S.C. (2023). A framework to estimate climate mitigation potential for US cropland using publicly available data. *J Soil Water Conserv*, 78, 193–206.

Nichols, J.D. & MacKenzie, D.I. (2004). Abundance estimation and Conservation Biology. *Anim Biodivers Conserv*, 27, 437–439.

Obembe, O.S. & Hendricks, N.P. (2022). Marginal cost of carbon sequestration through forest afforestation of agricultural land in the southeastern United States. *Agricultural Economics (United Kingdom)*, 53, 59–73.

Otto, C.R., Smart, A., Cornman, R.S., Simanonok, M. & Iwanowicz, D.D. (2020). *Forage and habitat for pollinators in the northern Great Plains—Implications for US Department of Agriculture conservation programs.*

Partners in Flight. (2024). *Avian Conservation Assessment Database*. *http://pif.birdconservancy.org/ACAD*.

Porensky, L.M. & Blumenthal, D.M. (2016). Historical wildfires do not promote cheatgrass invasion in a western Great Plains steppe. *Biol Invasions*, 18, 3333–3349.

Quinlan, G.M., Milbrath, M.O., Otto, C.R.V. & Isaacs, R. (2021). Farmland in U.S. Conservation Reserve Program has unique floral composition that promotes bee summer foraging. *Basic Appl Ecol*, 56, 358–368.

Quinlan, G.M., Miller, D.A.W. & Grozinger, C.M. (2023). Examining spatial and temporal drivers of pollinator nutritional resources: evidence from five decades of honey bee colony productivity data. *Environmental Research Letters*, 18.

Rao, M.N. & Yang, Z. (2010). Groundwater impacts due to conservation reserve program in Texas County, Oklahoma. *Applied Geography*, 30, 317–328.

Reeves, R.A., Pierce, C.L., Smalling, K.L., Klaver, R.W., Vandever, M.W., Battaglin, W.A., *et al.* (2016). Restored Agricultural Wetlands in central Iowa: Habitat Quality and Amphibian Response. *Wetlands*, 36, 101–110.

Rey Benayas, J.M. & Bullock, J.M. (2012). Restoration of Biodiversity and Ecosystem Services on Agricultural Land. *Ecosystems*, 15, 883–899.

Rey Benayas, J.M. & Bullock, J.M. (2015). Vegetation Restoration and Other Actions to Enhance Wildlife in European Agricultural Landscapes. In: *Rewilding European Landscapes*. Springer International Publishing, Cham, pp. 127–142.

Ritchie, H. & Roser, M. (2019). Half of the world’s habitable land is used for agriculture. *Our World in Data*.

Rosenberg, K. V., Dokter, A.M., Blancher, P.J., Sauer, J.R., Smith, A.C., Smith, P.A., *et al.* (2019). Decline of the North American avifauna. *Science (1979)*, 366, 120–124.

Ruan, L. & Philip Robertson, G. (2013). Initial nitrous oxide, carbon dioxide, and methane costs of converting conservation reserve program grassland to row crops under no-till vs. conventional tillage. *Glob Chang Biol*, 19, 2478–2489.

Sainju, U.M., Stevens, W.B., Caesar-TonThat, T. & Jabro, J.D. (2010). Land Use and Management Practices Impact on Plant Biomass Carbon and Soil Carbon Dioxide Emission. *Soil Science Society of America Journal*, 74, 1613–1622.

Saturday, A. (2018). Restoration of Degraded Agricultural Land: A Review. *J Environ Health Sci*, 4, 44–51.

Shaffer, J.A., Igl, L.D., Johnson, D.H., Sondreal, M.L., Goldade, C.M., Nenneman, M.P., *et al.* (2021). *The Effects of Management Practices on Grassland Birds-Grasshopper Sparrow (Ammodramus savannarum) The Effects of Management Practices on Grassland Birds Professional Paper 1842-GG*.

Sullivan, P., Hellerstein, D., Hansen, L., Johansson, R., Koenig, S., Lubowski, R.N., *et al.* (2004). The Conservation Reserve Program: Economic Implications for Rural America. *SSRN Electronic Journal*.

Swartz, T.M. & Miller, J.R. (2019). Managing farm ponds as breeding sites for amphibians: key trade-offs in agricultural function and habitat conservation. *Ecological Applications*, 29.

Tanner, E.P., Tanner, A.M., Fuhlendorf, S.D., Elmore, R.D., Davis, C.A. & Polo, J.A. (2021). Land enrolled in the Conservation Reserve Program supports roosting ecology of the lesser prairie-chicken. *Glob Ecol Conserv*, 32.

Troudet, J., Grandcolas, P., Blin, A., Vignes-Lebbe, R. & Legendre, F. (2017). Taxonomic bias in biodiversity data and societal preferences. *Sci Rep*, 7.

USDA-FSA. (2017). *CRP Years Enrolled by State Sep 2017*.

USDA-FSA. (2020). *The Conservation Reserve Program: A 35-Year History*.

USDA-FSA. (2024). *CRP Practices Library*. Available at: https://www.fsa.usda.gov/programs-and-services/conservation-programs/crp-practices-library/index. Last accessed 25 July 2024.

Vanbergen, A.J., Garratt, M.P., Vanbergen, A.J., Baude, M., Biesmeijer, J.C., Britton, N.F., *et al.* (2013). Threats to an ecosystem service: Pressures on pollinators. *Front Ecol Environ*.

Walston, L.J., Hartmann, H.M., Fox, L., Macknick, J., McCall, J., Janski, J., *et al.* (2024). If you build it, will they come? Insect community responses to habitat establishment at solar energy facilities in Minnesota, USA. *Environmental Research Letters*, 19.

Wauchope, H.S., Amano, T., Geldmann, J., Johnston, A., Simmons, B.I., Sutherland, W.J., *et al.* (2021). Evaluating Impact Using Time-Series Data. *Trends Ecol Evol*.

Whelan, C.J., Wenny, D.G. & Marquis, R.J. (2008). Ecosystem services provided by birds. *Ann N Y Acad Sci*.

White, E.R. (2019). Minimum Time Required to Detect Population Trends: The Need for Long-Term Monitoring Programs. *Bioscience*, 69, 26–39.

Wilson, M.W., Ridlon, A.D., Gaynor, K.M., Gaines, S.D., Stier, A.C. & Halpern, B.S. (2020). Ecological impacts of human-induced animal behaviour change. *Ecol Lett*, 23, 1522–1536.

Wu, J. & Weber, B. (2012). *Implications of a Reduced Conservation Reserve Program The Conservation Crossroads in Agriculture*.

Yang, Y., Loecke, T. & Knops, J.M.H. (2022). Surface soil organic carbon sequestration under post agricultural grasslands offset by net loss at depth. *Biogeochemistry*, 159, 303–313.

Yin, D., Wang, L., Zhu, Z., Clark, S.S., Cao, Y., Besek, J., *et al.* (2021). Water quality related to Conservation Reserve Program (CRP) and cropland areas: Evidence from multi-temporal remote sensing. *International Journal of Applied Earth Observation and Geoinformation*, 96.

Youngquist, M.B. & Boone, M.D. (2014). Movement of amphibians through agricultural landscapes: The role of habitat on edge permeability. *Biol Conserv*, 175, 148–155.

Zenone, T., Gelfand, I., Chen, J., Hamilton, S.K. & Robertson, G.P. (2013). From set-aside grassland to annual and perennial cellulosic biofuel crops: Effects of land use change on carbon balance. *Agric For Meteorol*, 182–183, 1–12.

Figure 1

Click here to access/download;Figure;Figure 1.tif

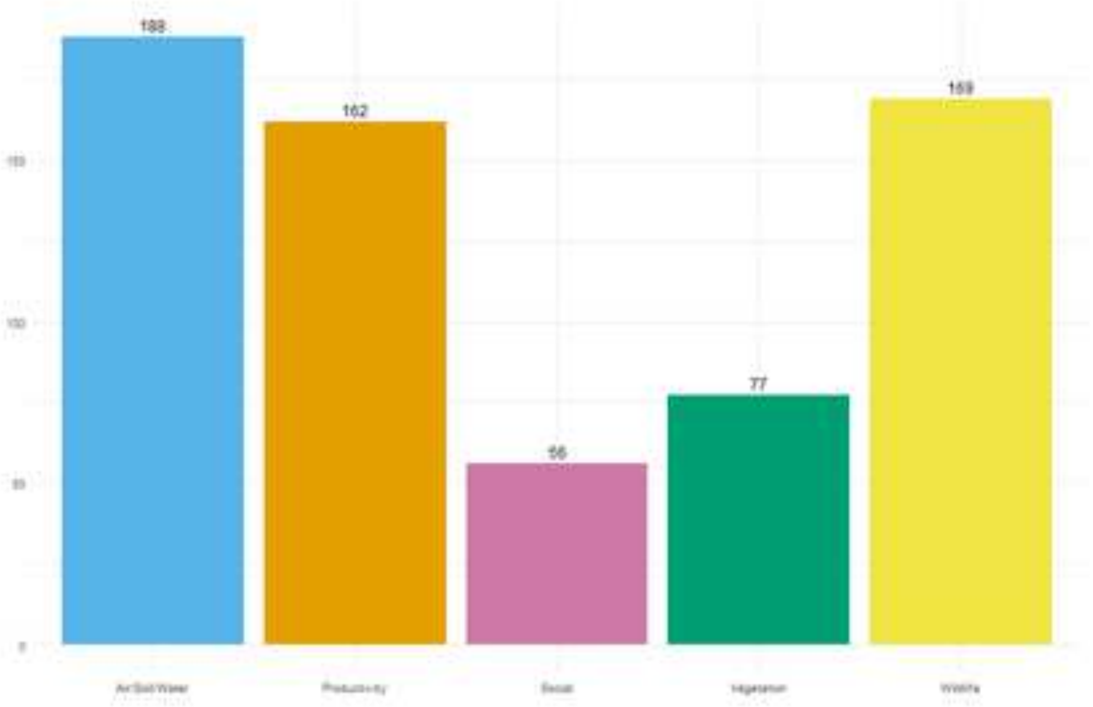

Figure 2

Click here to access/download;Figure;Figure 2.tif

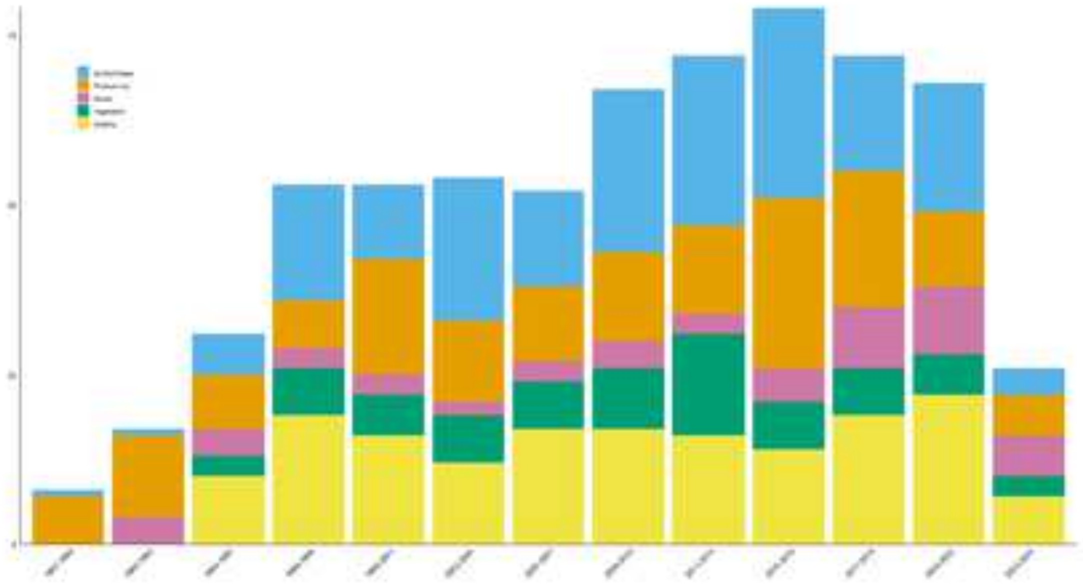

Figure 3

Click here to access/download;Figure;Figure 3.tif

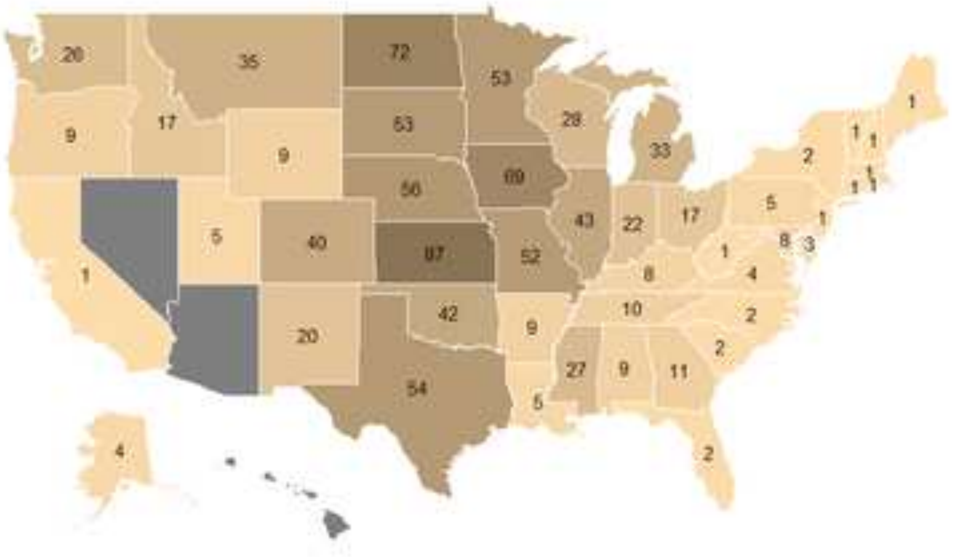

Figure 4

Click here to access/download;Figure;Figure 4.tif

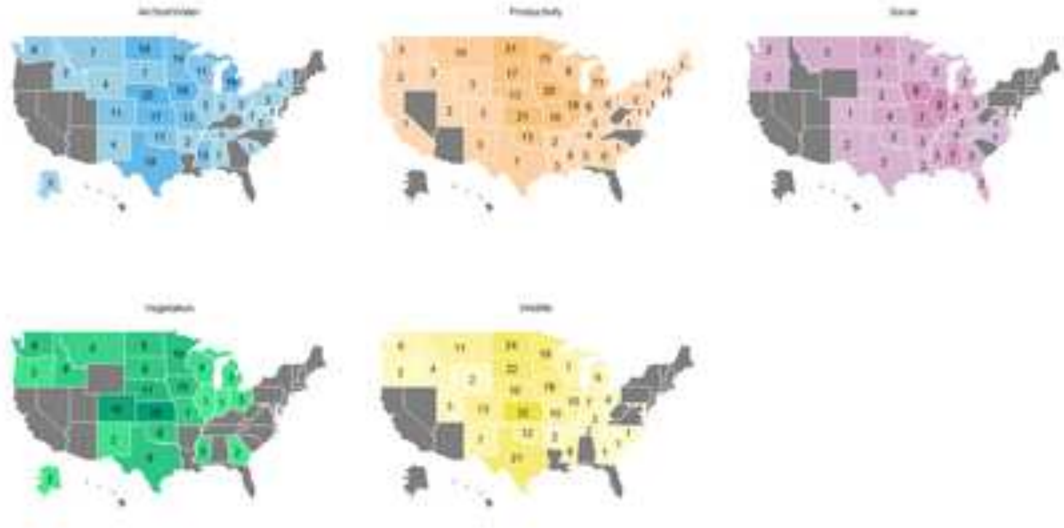

Figure 5

Click here to access/download;Figure;Figure 5.tif

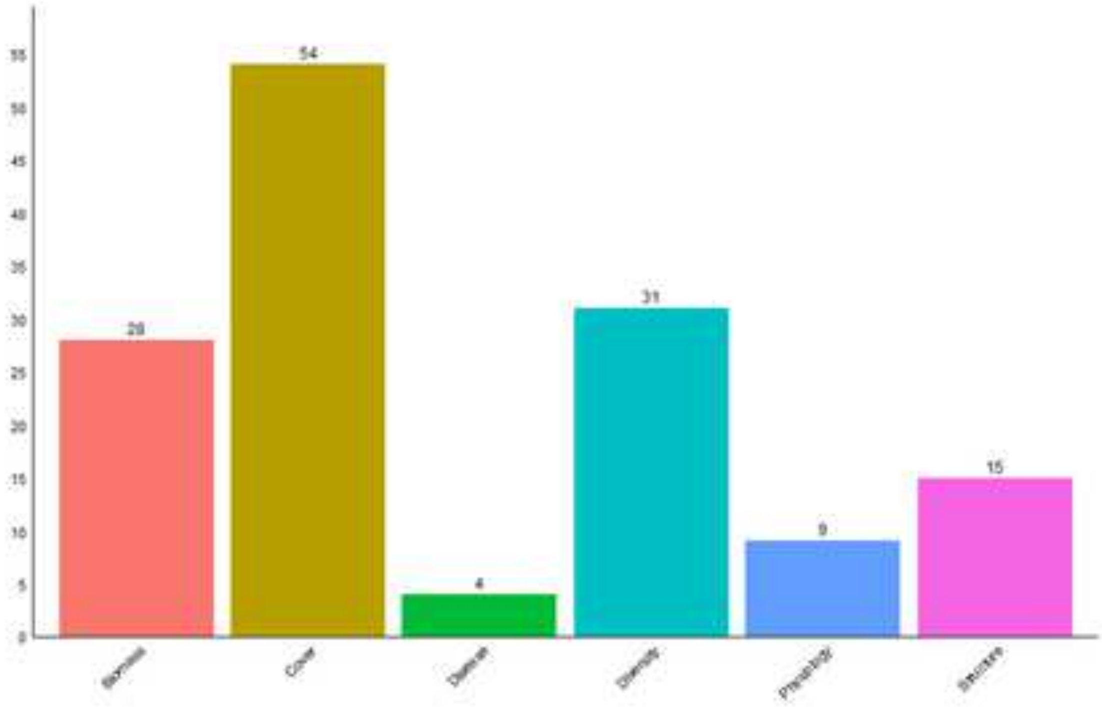

Figure 6 Click here to access/download;Figure;Figure 6.tif

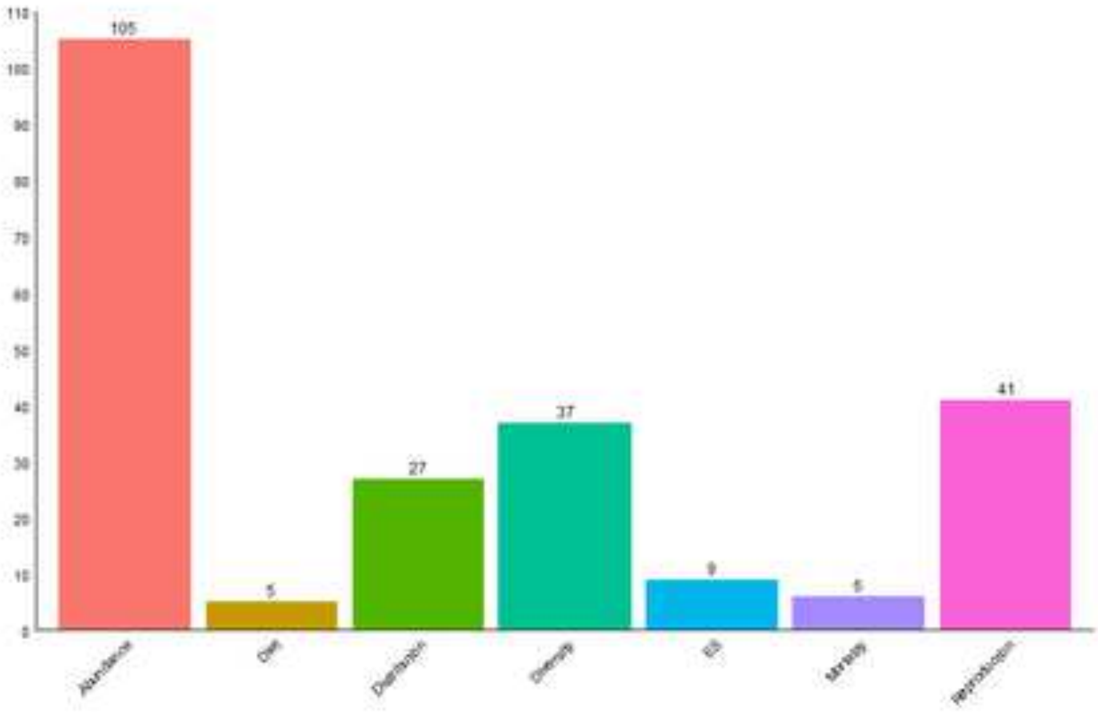

Figure 7

Click here to access/download;Figure;Figure 7.tif

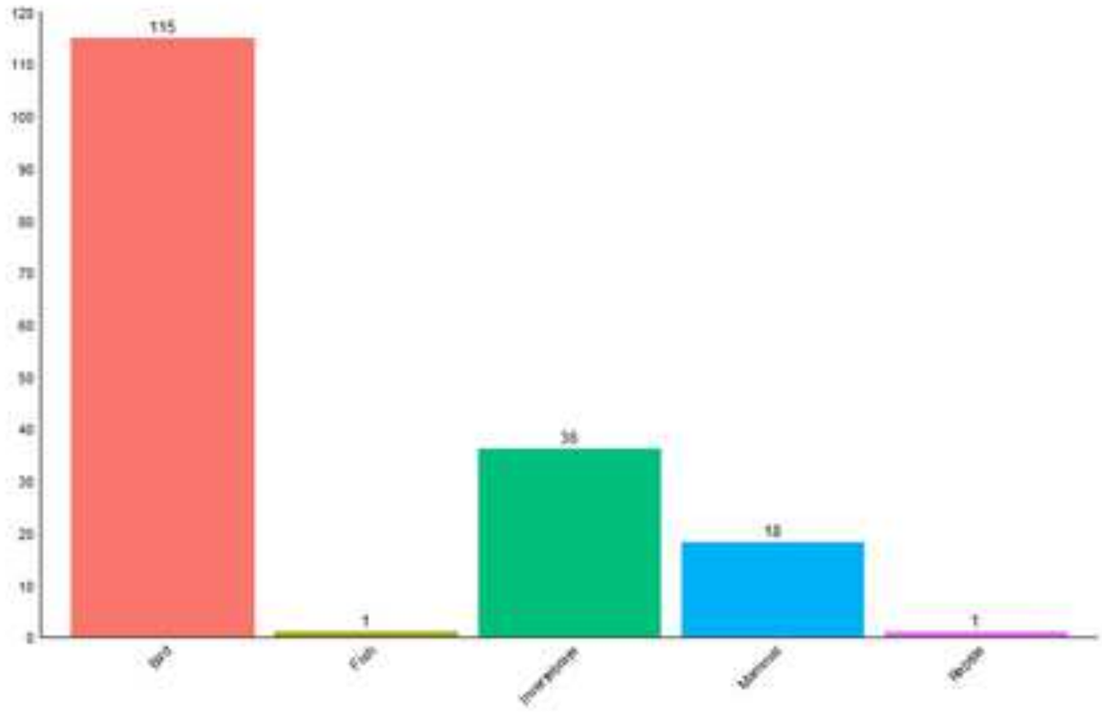

Click here to access/download
Supporting Information
Table S1.xlsx

Click here to access/download
Supporting Information
Table S1.docx

Supporting Information T3

Click here to access/download
Supporting Information
Table S2.csv

Click here to access/download
Supporting Information
PRISMA-ScR-Checklist.docx
