# Supplementary material for: Scoping review of the literature on outcomes of the conservation reserve program
Source: PLoS One. 2025 Aug 12;20(8):e0329962. doi: 10.1371/journal.pone.0329962 (PMC12342260; doi:10.1371/journal.pone.0329962)
Supplement: Attachment — Submitted filename: PlosOne Scoping review of CRP REVIEW.pdf [file pone.0329962.s006.pdf]

Greetings,

Thank you for the opportunity to review “Scoping Review and synthesis of the literature on the outcomes of the Conservation Reserve Program” by Nessel et al. I feel this is an interesting manuscript and an important topic; as it does a good job doing the tedious task of sorting through 40 years of literature (mostly focused on the last 25) to contribute to scientific understanding on what/where/and how Conservation Reserve Program (CRP) research is being applied.

Management decisions are guided by regional conservation views and the authors document an extensive literature search/meta-analysis using five major categories— studies of wildlife, vegetation, air/soil/water, productivity, and social aspects to highlight and better understand where additional research on CRP is needed.

Some thoughts/suggestions on the paper:

Throughout the manuscript the authors are inconsistent with their use of a comma after etc.

Line #34: I respect the difficulty separating the categories as many CRP studies are interrelated

Line #48: Why not include the U.S? Or forgo any mention of habitable land around the world all together in favor of U.S. habitable land, as this would be more topical to the paper.

Line #68: I don’t think “Management practices” is correct, these are conservation practices. Either change to “conservation” or just delete “management”.

Line #72: I think if the authors looked hard enough they could find evidence of CRP benefits dating back to the late 1980’s/early 1990’s, but since most of the literature is not electronically available from back then (which the authors do mention), it’s probably not too critical. But it would be interesting to find the first source that champions the benefits of CRP shortly after its establishment in 1985.

Line #99: I am a little shocked that after 40 years this is considered the first scoping review of CRP literature, but I have no evidence to show otherwise. I do, however, believe a large number of papers discuss the importance of continued research on the paper’s subject matter as very rarely does one research paper answer all of the questions. So I’m not sure it is fair to say future research on important aspects have not been emphasized in the literature. Maybe it has not been included in a scoping review, but on a case-by-case basis, I think the majority of discussion sections deal with the need for more research on the subject.

Line #129: Reiterating what I said earlier, it is a bit of a misnomer because a good portion of the papers written in the first 15 years of the CRPs existence may be excluded from your scoping review as some drastic changes happened to the landscape in those early years when tens of millions of acres went from row-crop to grassland. But this is more of a comment then a suggested edit. And I appreciate the authors make mention of the year 2000 as a point of reference (although they do frequently cite pre-2000 papers).

Line #153: Delete practice

Line #194: Average of 20 studies fitting the scoping review criteria, or of 20 studies in general?

Line #282: Was CREP included? I know the CRP is a vast program with many offshoots, but if one were to find a CRP study on amphibians, I would expect to look at CREP studies.

Line #282: I think bats are a bit of a stretch to be researched in CRP grasslands.

Line #322: Similar to amphibians and bats, I don’t think one would find, or expect to find much literature on the impacts of fish on general CRP (if that was the focus) as it is primarily a grassland program. Although one could valiantly argue that fish could be negatively affected by sediment runoff or positively affected by buffering of streams.

Line #425: Yes and no. I agree, that further research is needed, but this has to do with how programmatic changes (e.g., more frequent emergency use) and climatic changes (e.g., more frequent droughts) are affecting the role of CRP. Forty years of literature on the CRP has done an adequate job of explaining direct and indirect benefits born from the program.

Line #453: Good point. Abundance alone can misleading.

Line #484: Be consistent with capitalization. LPC and GPC are inconsistent

Line #525: delete (CRP)

Table S1: This information is old. Update it using this website: Conservation Reserve Program (CRP) Statistics | Farm Service Agency

The CRP has upwards of 40-50 practices, depending how you look at it. My gut assumption was there were many more studies on CRP and this scoping review failed to find them. But the authors clearly define in the methods the sideboards used to narrow the scope and focus. I appreciate the amount of work dedicated to this review, as I am familiar with how labor intensive these efforts can become. Because of the vastness of the studies reviewed I feel the authors were successful in making every attempt to do a comprehensive review of the literature.

I will confirm though, as evidence of value, that I will incorporate understanding gleaned from the views and impacts resultant from this manuscript into expanding future research into underrepresented CRP lands.
