# Supplementary material for: Scoping review of the literature on outcomes of the conservation reserve program
Source: PLoS One. 2025 Aug 12;20(8):e0329962. doi: 10.1371/journal.pone.0329962 (PMC12342260; doi:10.1371/journal.pone.0329962)
Supplement: Attachment — Submitted filename: Response to Reviewers.docx [file pone.0329962.s007.docx]

April 24, 2025

Dear Dr. Pagano,

We thank you for giving us the opportunity to respond to the thoughtful remarks of the two reviewers and re-submit our manuscript to *PLOS ONE*. We find the comments very helpful, fair, and constructive. We have put a considerable effort into fully revising our manuscript and carefully addressed all the concerns and recommendations. We hope you agree that our manuscript has benefited considerably from those improvements and it is now suitable for publication in *PLOS ONE*.

Regarding Reviewer 1’s comments, most of these asked us to provide additional synthesis of the literature in the form of summaries of the findings of CRP studies within different areas of inquiry (e.g., a synthesis about what the literature says about effects of CRP on soil nutrients, wildlife, etc.). Although we provided thorough responses to all of Reviewer 1’s comments, we decided not to make revisions of this sort because the objective of a scoping review is to characterize trends, emphases, and gaps in what has and has not been studied in a field of research, and not to qualitatively summarize the findings of that literature. We agree that a narrative review or meta-analysis study with the goal of synthesizing the literature in this way would be extremely useful, and indeed we are currently finalizing a meta-analysis paper synthesizing the effects of CRP on wildlife outcomes. However, providing this type of synthesis along with our scoping review results is intractable; to address these comments fully, we would arguably need to double the length of the Discussion section, or even draft a different type of review paper. We hope this rationale for maintaining the scope of the paper as a scoping review, and please note that, to address the reviewer’s concerns, we did add clarification about the intended scope of the paper in the Methods section. Below we provide our detailed responses to the reviewers’ comments. Our replies are in bold font. The line numbers we report in our response correspond to the unmarked version of our revised paper without tracked changes.

We look forward to your assessment of this revised version of the manuscript.

Sincerely, and on behalf of my co-authors,

Mark Nessel

Reviewer #1:

The authors conducted a systematic review of the effects of Conservation Reserve Program in USA. They systematically searched for studies dealing with Soil/vegetation/wildlife/social and other aspects that were affected by CRP. They also conducted spatial analyses to show which states participating in the program have conducted studies to assess the effects of CRP. The study was done well and summaries have been provided on how many studies were conducted on different habitat/organismal factors affected by CRP. However, the authors did not provide good syntheses in the Discussion. For example, there is good summaries on how many studies covered soil improvement and from which State(s), but there are limited or no syntheses extracted from these studies as discussion points. The same is true of vegetation, wildlife, social studies etc. I feel the paper would improve a lot more if such information is summarized and synthesized from the substantial numbers of papers to enrich the discussion. I suggest major review and i have appended a marked up version of the manuscript where I propose these changes need to be incorporated.

**We thank the reviewer for their comments. Because this is a scoping review and not a meta-analysis we intentionally did not extract or synthesize the results from our studies. A scoping review isn't a review of what the literature has found but instead is a review of what the literature has and hasn't evaluated. As such, we only report on things we could tally or measure from the papers we extracted, and avoid editorializing or selecting examples that could be misinterpreted by a reader as an overall effect when it is, in fact, a single anecdotal example from our database. As an example, the Grasshopper Sparrow is the most common bird in our database, appearing in 50 studies. If we pick any single study to highlight we might find CRP to have a positive, negative, or non-significant effect, in the specific habitat, and on whichever metric the authors of the studies looked at.**

**In response to this comment, and in an effort to further emphasize the above point, a sentence was added to the *Study categorization* section of the methods: “Because this is a scoping review and not a meta-analysis or narrative review, and therefore, because our objective was to characterize trends, emphases, and gaps in what has and has not been studied with regard to CRP effects on wildlife, we intentionally did not extract or synthesize the results from our studies, and instead only focused on what the literature did and did not evaluate.” (See lines 149 to 152). Also, to avoid confusion about the scope of the paper, we deleted “synthesis” from the title and from other locations within the paper.**

Line 69: Spell out what CP (Conservation Practice) means for international audiences when first mentioned.

**We have expanded the sentence to spell out what these practices are. The sentence now reads: “These practices, which are indicated by “CP” followed by a number, include "Establishment of Permanent Native Grasses" (CP2), "Riparian Buffers" (CP22), and "Habitat Buffers for Upland Birds" (CP33) (USDA-FSA 2024).”**

Line 389: A broader synthesis on how soil, water and other aspects of habitat quality were improved is needed. Which soil parameters were improved (N? P? pH?) ? Which air quality measures were improved? The social aspects should be separated out and a synthesis needs to be provided on broadly which social aspects were improved.

**As described in greater detail above, synthesizing the results of the studies collected is outside the scope of this type of review paper.**

Line 427: I would have wanted more discussion/synthesis on the effects of the CRP on wildlife. Specific examples such as "species A increased due the program in the State X" or "community diversity or overall biomass of a species increased in riparian habitats"... this would make the discussion more informative compared to whether 'n' number of studies addressed issues relating to biomass and diversity.

**As described in greater detail above, synthesizing the results of the studies collected is outside the scope of this type of review paper.**

Line 432: Ok. But what do some of these short term studies driven by graduate students demonstrate. Some synthesis would be essential

**Thank you for this comment. In order to clarify what types of conclusions are possible with short term research we have added the sentence: “Although short-term studies can provide invaluable insights about associations between CRP and outcomes like wildlife and vegetation abundance, occurrence, species diversity, and reproductive rates, long-term and large-scale studies are important for accurately measuring ecological trends and capturing lag effects that might not manifest until years after management is applied.” (See lines 437 to 441).**

Line 458: So, make this more informative by picking some examples of species that were increased due to either land management acitivies but was mis-interpretted as a those associated with CRP.

**This is another similar comment about synthesis as those above, and please see our above, more-detailed responses.**

Line 464: I agree that more emphasis needs to be placed on other taxa. However, what do some of the 68% of biased studies show? Are some of the non-native species primarily benefitted or are some of the common species increased? Please provide specific examples and sythesize the information about overall conclusions of trends made by the authors of the study.

**Again, see the above more-detailed responses regarding literature syntheses; providing all of this requested information would greatly increase the length of the discussion, and arguably would need to be done in an entirely separate narrative review paper.**

Line 480: For example? which non-listed species of the ACAD watch list were considered?

Line 485: Ok

**We don’t believe this comment requires a specific revision. After the text highlighted by the comment, we continue to list bird species collected in our studies as well as report all of the bird species from our collected studies in Table S3. We interpret the “OK” left by the reviewer a few lines down, as them agreeing with this interpretation.**

Line 485: What is the overall nature of the benefit to these species: did their densities increase? Did they have higher reproductive success?

**Synthesizing the results of the studies collected is outside the scope of this review.**

Reviewer #2:

Greetings,

Thank you for the opportunity to review “Scoping Review and synthesis of the literature on the outcomes of the Conservation Reserve Program” by Nessel et al. I feel this is an interesting manuscript and an important topic; as it does a good job doing the tedious task of sorting through 40 years of literature (mostly focused on the last 25) to contribute to scientific understanding on what/where/and how Conservation Reserve Program (CRP) research is being applied.

Management decisions are guided by regional conservation views and the authors document an extensive literature search/meta-analysis using five major categories— studies of wildlife, vegetation, air/soil/water, productivity, and social aspects to highlight and better understand where additional research on CRP is needed.

Some thoughts/suggestions on the paper:

Throughout the manuscript the authors are inconsistent with their use of a comma after etc.

**Because "etc." was only used once in the manuscript, we were unclear about how to address this comment. We reviewed comma use after other abbreviations (e.g.) and tried to maintain consistency, but would be happy to further address this issue with additional clarification**

Line #34: I respect the difficulty separating the categories as many CRP studies are interrelated

**Thank you for this comment, which we do not interpret as requesting a specific revision.**

Line #48: Why not include the U.S? Or forgo any mention of habitable land around the world all together in favor of U.S. habitable land, as this would be more topical to the paper.

**We have added information about US agricultural land to the statement. It now reads: “The Food and Agriculture Organization of the United Nations estimates that agricultural land makes up 48 million km2, or about 45% of all habitable land on earth (FAO 2024; Ritchie & Roser 2019), and in 2022, 45.1% of U.S. land area was estimated to be devoted to agricultural use (World Bank 2025).” (See lines 44 to 48)**

Line #68: I don’t think “Management practices” is correct, these are conservation practices. Either change to “conservation” or just delete “management”.

**We have removed management from the sentence. It now reads: “Sharing a common theme of perennial cover, the broad range of practices…”** **(See lines 64 to 65)**

Line #72: I think if the authors looked hard enough they could find evidence of CRP benefits dating back to the late 1980’s/early 1990’s, but since most of the literature is not electronically available from back then (which the authors do mention), it’s probably not too critical. But it would be interesting to find the first source that champions the benefits of CRP shortly after its establishment in 1985.

**Thank you for this comment, which we do not interpret as requesting a specific revision.**

Line #99: I am a little shocked that after 40 years this is considered the first scoping review of CRP literature, but I have no evidence to show otherwise. I do, however, believe a large number of papers discuss the importance of continued research on the paper’s subject matter as very rarely does one research paper answer all of the questions. So I’m not sure it is fair to say future research on important aspects have not been emphasized in the literature. Maybe it has not been included in a scoping review, but on a case-by-case basis, I think the majority of discussion sections deal with the need for more research on the subject.

**Thanks for catching us on perhaps overstepping with our statement of novelty of the research. We have changed the language of this sentence in order to not overstate the novelty. The sentence now reads: “With this review, we seek to inform and guide future research on important aspects of CRP that have to this point not received formal empirical evaluations in the literature.”**

Line #129: Reiterating what I said earlier, it is a bit of a misnomer because a good portion of the papers written in the first 15 years of the CRPs existence may be excluded from your scoping review as some drastic changes happened to the landscape in those early years when tens of millions of acres went from row-crop to grassland. But this is more of a comment then a suggested edit. And I appreciate the authors make mention of the year 2000 as a point of reference (although they do frequently cite pre-2000 papers).

**Thank you for this comment, which we do not interpret as requesting a specific revision.**

Line #153: Delete practice

**All text that said “CP Practices” has been changed to “Conservation Practices”**

Line #194: Average of 20 studies fitting the scoping review criteria, or of 20 studies in general?

**Text was added to this sentence to clarify the statement. The new sentence reads: “During the 30-year period between 1994 and 2023 an average of 20 studies examining the effects of CRP were published per year ranging from 11 studies in 1995 to 36 studies in 2016.”**

Line #282: Was CREP included? I know the CRP is a vast program with many offshoots, but if one were to find a CRP study on amphibians, I would expect to look at CREP studies.

**We did not explicitly search for CREP (see search terms in the methods), but CREP studies were captured in our search, and included in our database. We had already acknowledged in the *Study Limitations* section of the discussion that there are some potential problems that might arise when trying to collect every paper of interest; however, in response to this comment we also revised to mention that our review seemed to also capture studies on very closely related offshoot programs, like CREP: “Because of this we were able to capture some studies that evaluated sub-programs, such as the Conservation Reserve Enhancement Program (CREP) (e.g., Manley & Mathias 2017; Wentworth et al. 2010); however, our database is not an exhaustive search of these offshoots” (See lines 514 to 517)**

Line #282: I think bats are a bit of a stretch to be researched in CRP grasslands.

**Our focus was not only on CRP grasslands, but all land enrolled in the CRP program. Further, bats occur in airspace over grasslands and are a species of interest in many agricultural studies. We believe it worth pointing out that this important species was totally absent from the CRP studies we collected.**

Line #322: Similar to amphibians and bats, I don’t think one would find, or expect to find much literature on the impacts of fish on general CRP (if that was the focus) as it is primarily a grassland program. Although one could valiantly argue that fish could be negatively affected by sediment runoff or positively affected by buffering of streams.

**In the same way as bats, fish occupy freshwater bodies adjacent to CRP land, and are the subject of many studies examining the effect of agricultural practices on their ecology. We think it’s worthy to highlight their complete absence from the CRP literature that we were able to collect, even if we don’t expect them to be the preponderance of the study taxa.**

Line #425: Yes and no. I agree, that further research is needed, but this has to do with how programmatic changes (e.g., more frequent emergency use) and climatic changes (e.g., more frequent droughts) are affecting the role of CRP. Forty years of literature on the CRP has done an adequate job of explaining direct and indirect benefits born from the program.

**Text was added to the sentence to clarify our statement: “Further research that continues to evaluate the wildlife and vegetation-mediated ecosystem services provided by CRP, especially how these services are affected by implementation of programmatic changes will be crucial to further increasing understanding of the role of CRP in providing benefits to the environment and humans.”** **(See lines 429 to 432)**

Line #453: Good point. Abundance alone can misleading.

**Thank you for this comment, which we do not interpret as requesting a specific revision.**

Line #484: Be consistent with capitalization. LPC and GPC are inconsistent

**All of the capitalization on the bird common names have been standardized in the text.**

Line #525: delete (CRP)

**Corrected**

Table S1: This information is old. Update it using this website: Conservation Reserve Program (CRP) Statistics | Farm Service Agency

**We are aware that this is not the most up to date enrolment numbers for CRP. As this study is looking back on decades of literature published, we wanted to pick a year closer to when the peak of these publications were being made. To emphasize this, text was added to the methods: “The amount of land enrolled in the program fluctuates by year, so data from 2017 was chosen as it was the year after the greatest number of CRP studies meeting our study inclusion criteria was published.” (See lines 158 to 160) to explain that we chose data from the year after the greatest number of CRP studies meeting our study inclusion criteria was published. Also Table S2 was mislabeled as Table S1 within the file. This has been fixed,**

The CRP has upwards of 40-50 practices, depending how you look at it. My gut assumption was there were many more studies on CRP and this scoping review failed to find them. But the authors clearly define in the methods the sideboards used to narrow the scope and focus. I appreciate the amount of work dedicated to this review, as I am familiar with how labor intensive these efforts can become.

Because of the vastness of the studies reviewed I feel the authors were successful in making every attempt to do a comprehensive review of the literature.

I will confirm though, as evidence of value, that I will incorporate understanding gleaned from the views and impacts resultant from this manuscript into expanding future research into underrepresented CRP lands.

**Thanks for your supporting words. We did not interpret anything in these 3 related comments as being requests for revisions.**
