# Supplementary material for: Scoping review of the literature on outcomes of the conservation reserve program
Source: PLoS One. 2025 Aug 12;20(8):e0329962. doi: 10.1371/journal.pone.0329962 (PMC12342260; doi:10.1371/journal.pone.0329962)
Supplement: Attachment — Submitted filename: Response_to_Reviewers_auresp_2.docx [file pone.0329962.s008.docx]

June 20, 2025

Dear Dr. Pagano,

We thank you for the opportunity to submit this second revised version of our manuscript to PLOS ONE. We appreciate the continued engagement from the reviewers and editor, and we have carefully addressed the additional comments raised during this round of review.

As a reminder, this revision follows a previous set of reviewer comments, which we addressed in full in our initial resubmission, and received no further feedback. We understand that this current round reflects further input from an additional reviewer, and we have treated these as a distinct set of revisions.

In this revision, we made several modest changes to the manuscript to improve clarity and accuracy. These include: clarifying our interpretation of temporal patterns in the literature; refining figure formatting and labeling (including annual publication counts and font adjustments); and incorporating small textual clarifications to address reviewer concerns around terminology and interpretation.

We hope these changes further strengthen the manuscript and demonstrate our responsiveness to the feedback. Below we provide our detailed responses to the reviewers’ comments. Our replies are in bold font. The line numbers we report in our response correspond to the unmarked version of our revised paper without tracked changes.

Sincerely, and on behalf of my co-authors,

Mark Nessel

Reviewer #3: Overall, understanding research emphases and gaps in the Conservation Reserve Program (CRP) literature is an important question for continental conservation of cropland modified ecosystems. The article presents a thorough scoping review of the primary literature for research on the CRP. The objectives of the study were to 1) characterize spatial and temporal trends in CRP research, 2) categorize CRP research by type, including studies of wildlife; vegetation; air, soil, and water; and more human-focused aspects, and 3) evaluate detailed characteristics of studies that focus on responses of wildlife and vegetation to CRP. The findings indicate most studies are concentrated in the Great Plains, leaving regions such as the Pacific Northwest underrepresented. For wildlife-related outcomes of CRP, birds dominated the literature, while research on other taxa such as fish, reptiles, and amphibians are sparse.

The main considerations identified for improving the manuscript include 1) clarifying concepts and terminology for wildlife habitat relationships, 2) revising claims about lack of temporal trends not supported by data, 3) providing a caveat or discussion on the sensitivity of conclusions to double counting frequency of studies, and 4) discussing the importance of disturbance and management for species-specific habitat suitability of CRP.

**Thank you for these constructive comments and the positive appraisal. We respond to all of these major considerations below.**

Specific comments

Lines 43 – 49: Although it is appropriate to discuss agriculture in general to introduce the study, agriculture can refer to animal production, forestry, soil science, etc. Consider framing study around conservation problems associated with farming and cultivated cropland, resulting in grassland loss and associated habitat loss and fragmentation for grassland species.

**Thank you for this suggestion. In order to focus on habitat loss associated with farming we changed the sentence to read “In particular, land use changes associated with agricultural crop production are contributing substantially to biodiversity loss (Caro et al. 2022; Jaureguiberry et al. 2022).” [lines 47-48]**

Lines 54 – 56: Consider mentioning Farm Bill Legislation as the funding mechanism.

**We added a clause to the sentence indicating the Farm Bill administers the CRP program.**

Lines 100 – 185: No quantities to be summarized or statistical methods were described making it difficult to understand how data from the literature review will be used to address the study objectives. Consider adding a sentence or two to describe the summaries used to draw conclusions from the review.

**In order to clarify our approach for generating descriptive summaries in support of addressing our objectives, we added a sentence to the methods: “To address the objectives of this scoping review, we generated total counts of studies and calculated proportions of studies in different categories in order to identify trends and gaps in the literature.” [lines 152-154]**

Lines 141 – 143: Consider adding a sentence to indicate that summaries were calculated with some studies counted more than once. This disclaimer appears in the figure captions, but the issue of double counting studies in the summaries must be stated in the article. This may be yanking around results based on summarizing the frequency of studies. Even a scoping review requires an evaluation that claims are supported by the data.

**We added some text to further clarify how we counted studies that belonged under multiple categories: “As with the study categories above, many of these categories were not mutually exclusive, meaning one study could contribute multiple data points (e.g., a study that evaluated multiple conservation practices). However, because such studies were only included in descriptive summaries of total counts of studies or to calculate proportions of total studies (e.g., X% of studies examining bird species),any individual study was only counted once for any single metric that we present” [lines 161-166]**

Line 145: Consider a revision to improve precision of the concept for “wildlife habitat” used throughout the article. Because habitat is a species-specific concept, the concept of general “wildlife habitat” does not exist in wildlife ecology (Hall et al. 1997, Johnson 2007). In a scoping review, it is important to clarify concepts for the research area, and use of a term such as “wildlife habitat” incorrectly implies that CRP functions equally as habitat across species when habitat requirements for CRP vegetation is specific and unique for individual species.

**We appreciate the reviewer’s point and agree that “habitat” is most accurately defined in species-specific terms, as emphasized by the cited literature. We recognize the risk of overgeneralizing this term in ecological writing and have carefully reviewed our manuscript with this in mind.**

**As a scoping review, our goal is to synthesize and describe the findings of individual studies, each of which typically defines and evaluates habitat at the species level. When we refer to “wildlife habitat,” we are not suggesting a universal or undifferentiated habitat across all species, but rather using a shorthand to refer to the various species-specific habitats that individual studies have identified in relation to CRP. Our synthesis encompasses these distinct definitions rather than collapsing them into a single concept.**

**To clarify this in the manuscript and address the concern:**

**We have revised the term “wildlife habitat” where appropriate to provide greater precision or contextual clarity.**

**At the first mention of “wildlife habitat” in the Introduction (Line 71), we have added a parenthetical note acknowledging the species-specific nature of habitat and clarifying our intended use of the term in the paper. The revised sentence reads:**

**“These benefits include providing wildlife habitat (acknowledging that habitat is a species-specific concept, we use ‘wildlife habitat’ as a shorthand for the variety of species-specific habitats reported in the literature)” [lines 71-73]**

**We hope this clarification and the accompanying edits throughout the manuscript appropriately address the concern while preserving clarity and consistency in the narrative.**

Table 1: Was species richness used as an indication of diversity? If so, consider adding species richness to the diversity examples item.

**Species richness was included in our Diversity group. It has been added to the examples in Table 1 to indicate this.**

Table 1: Abundance is listed as a sperate row, but density, a measurement of abundance, is listed under Distribution. Consider adding a rational for having the same metric listed on different rows.

**Density is counted in the Abundance group. We use the word density in the description of the Distribution group. In order to alleviate this confusion the text was changed in the Distribution description to not include the word density.**

Figure 1: Consider adding figure axis titles, and fonts large enough to be read. Consider removing incomplete data for year 2024 from the figure because it is not comparable to the other time occasions.

**We appreciate the suggestion. We have increased the font size in all bar graphs (Figures 1, 2, 5, 6, and 7) to improve legibility. We also added consistent axis labels across all bar graphs. We believe the reference to 2024 belongs to Figure 2 (which displays numbers of studies for different publication years), not Figure 1. Accordingly, we removed the 2024 data from Figure 2 and clarified the presence of two 2024 studies in the figure legend instead. We also note that font scaling often depends on figure sizing during publication and may display differently depending on screen size and output resolution. Previous reviewers did not raise font legibility as an issue, but we are happy to work with the editor during copyediting to ensure the figures display as clearly as possible.**

Figure 2: Consider adding axis titles, and fonts large enough to be read. Consider labelling the bars with the numbers of studies so that the readers can decide if there is a trend in the number of studies over time.

**We updated all bar graphs to use a consistent y-axis title ("Number of Studies"). We retained the omission of x-axis titles since the category or year labels are already shown on the axis. We also added numeric labels above each bar in Figure 2 showing the total number of studies published each year.**

Figure 4: Consider making the headings large enough to be read

**Thank you. As noted above, we increased the font size for all bar graphs to improve clarity.**

Figure 5: Consider adding axis titles, and fonts large enough to be read

**We increased the font size of bar labels and axis text in all bar graphs and standardized the y-axis title as "Number of Studies," as described above.**

Lines 215 – 216: Consider quantifying in terms of a proportion or percentage

**We added text to clarify the exact states along to the 98^th^ meridian and report the exact number of studies conducted there: “The largest number of studies have occurred in states in** **the central part of the U.S.; specifically, 46% (n =230) of studies were in states along the 98th meridian** **(i.e., North Dakota, South Dakota, Nebraska, Kansas, Oklahoma and Texas), with most of the studies in our dataset occurring in Great Plains states (Figure 3).” [lines 226-229]**

Lines 218 – 219: Consider providing a correlation for the relationship

**We appreciate the reviewer’s suggestion to provide a correlation between CRP enrollment and the number of studies conducted in each state. However, after consideration, we opted not to conduct a formal correlation analysis for a few reasons. First, CRP enrollment is a dynamic variable that changes annually, while publication dates of CRP studies do not necessarily align with the specific year(s) of CRP data reported in those studies. As a result, a direct correlation between CRP acreage and study count across 50 states using a single year of enrollment data would provide limited interpretability.**

**To address this issue in a consistent and transparent manner, we used enrollment data from 2017, the year following the peak in study publication, as a fixed reference point. We then provided a quantitative summary: the 10 states with the most CRP acreage in 2017 accounted for 64% of studies with state-level data, averaging 54 studies per state. We also explicitly discussed deviations from this trend (e.g., Washington had fewer studies than expected given its acreage, while Wisconsin and Michigan had more).**

**We believe this descriptive approach provides meaningful context for understanding spatial research trends without overstating the strength or consistency of the relationship, and we have clarified this rationale in the manuscript.**

Line 251: See above, questionable use of terms “wildlife habitat” and “habitat quality”

**See above discussion about the use of the term “wildlife habitat”**

Figure 7: The figure is out of sequence. The figure needs axis titles, and fonts large enough to be read.

**Thank you for this comment. We reviewed the manuscript and confirmed that Figure 7 is cited in sequence following Figure 6. It is possible this comment was related to figure formatting during the review process. Nevertheless, we have double-checked the figure order to ensure accuracy. We also addressed the accompanying comments by updating axis titles and improving font sizes for clarity.**

Lines 319 – 320: Consider a revision to justify this sentence. Figure 2 indicates the number of studies increased over time.

**We appreciate the reviewer’s suggestion and agree that the original sentence could be misinterpreted as a statistical inference rather than a coarse summary of the literature timeline. To address this, we have revised the sentence to avoid implying a formal test of temporal variation and instead present a descriptive summary of the publication record. The updated text reads: “The published research on CRP has been relatively consistent temporally despite declines in enrolment” [lines 332-333]**

Lines 329 – 330: How was this determined? Consider discussing the apparent increase in the number of studies shown in Figure 2. A simple glm of apparent frequencies from Fig. 2, shows a large increase in the number of studies over time, and more evidence for a non-linear threshold increase over time

**Thank you for this observation. We agree that a formal statistical model could be used to assess trends in publication frequency over time. However, as our scoping review was not designed to evaluate statistical trends, we avoid quantitative inferences and instead describe the pattern as it appears in the updated version of Figure 2, which now includes annual (rather than binned) counts. To further clarify our intent, we revised the paragraph to remove references to “consistency”: “There were no major year-over-year changes in the type or number of studies published about CRP since the program’s inception in 1985. Beginning in 1994 we observed no multi-year trends in the number of published studies in all 5 categories.” [lines 342-344]**

Lines 330 – 332: The annual trend in the proportional change between study types is a different question than over all change over time. Consider providing some evidence for “relative consistent number of studies. This requires some sort of statistical support. For example, one could take the proportion for each type, and then test for correlation with year. That would probably show the same as the over all increase for Air/Soil/Water, Vegetation, and Wildlife, but possibly no increase over time for Productivity or Social.

**We agree that the distinction between the total number of studies and the proportional distribution across study types is important. In our analysis, we focused on describing the absolute number of studies per category and how those varied across time, rather than testing whether proportions shifted statistically. This is especially important as some studies would count under multiple categories making interpretation difficult. We have clarified this distinction in the text and avoided using “relatively consistent” to describe trends without statistical support.**

Figure 6: The figure needs axis titles, and fonts large enough to be read

**As with the other bar graphs, we increased font sizes and added axis titles to Figure 6.**

Lines 352 – 357: Run-on sentence. Consider discussing the primary reason for studying CRP in regions with high cultivated cropland is to understand the role of CRP to address habitat loss from grassland conversion, something not touched on in the introduction.

**Thank you for this helpful suggestion. We revised the sentence to improve clarity and added a statement acknowledging the disproportionate importance of CRP in regions with significant grassland loss (as noted in response to the above reviewer comment, we also revised a line in the introduction to better frame this paper within the context of grassland loss through agricultural crop production). The revised paragraph now reads:**

**“Identifying the factors driving these regional patterns is beyond the scope of this study. However, these spatial trends may relate to two key factors. First, the number of studies is likely related to the population of researchers available to study CRP in universities and other research institutions within each state. Second, CRP lands often represent a relatively large share of remaining grassland cover in heavily cultivated regions such as the Corn Belt and Great Plains, making CRP especially important for supporting habitat for various wildlife species and a focal point for conservation research.” [Lines 365-371]**

Line 361 – 365: Consider adding a sentence to discuss lack of disturbance as the reason why CRP may not provide habitat for the Grasshopper Sparrow in the eastern part of its range. In addition, the disturbance gradient for Grasshopper Sparrow also occurs within the Great Plains, and yet CRP is still an important conservation strategy for addressing habitat loss from grassland conversion for this species.

**Given our broad objective to characterize the CRP literature, we hesitate to get further into “the weeds” regarding the relevance of CRP to particular species. However, in order to address this broader point reflecting the reviewer’s concern (but without further dwelling solely on the Grasshopper Sparrow), we added the sentence “Thus, studies are needed that evaluate the degree to which CRP provides the required habitat conditions for such species across their entire ranges.” [Lines 379-380]**

Lines 371 – 382: Consider conservation goals to balance crop yields with the other CRP biophysical and biodiversity goals (Butsic et al. 2020).

**We appreciate this suggestion and agree that a key challenge—and opportunity—for CRP is balancing land retirement and ecological restoration goals with the realities of agricultural production. To acknowledge this important point, we have added the following sentence to the end of the paragraph discussing farm-level and stakeholder responses to CRP:**

**“This body of research, including studies of the human dimensions of CRP, has underscored the broader challenge of balancing conservation and land restoration goals of CRP with the other factors that influence stakeholder decisions about land use in agricultural landscapes (Butsic et al. 2020).” [Lines 398-401]**

Line 396: See above, no such thing as “wildlife habitat”

**See above discussion about the use of the term “wildlife habitat”**

Line 413: Consider revising this sentence or the previous conclusions lines 319 – 320 for no trends in the data over time

**The sentence was altered to highlight the prominence of wildlife-focused research in the CRP literature, not a directional change over time. “Wildlife in particular have become a larger priority in the CRP program over time (https://www.nrcs.usda.gov/ceap/wildlife), as reflected by the large proportion of CRP studies focused on wildlife outcomes and by the establishment of newer CRP practices, starting in the mid-2000s, that allow stakeholders to directly curate lands to benefit ecologically important wildlife species (CP38) including beneficial birds (CP33, CP37) and pollinators (CP42) (USDA-FSA 2024).” [Lines 431-436]**

Line 416 – 417: Consider rephrasing this sentence, habitat is a species-specific concept, CRP does not generally support wildlife habitat, each species has a different habitat relationship with CRP vegetation features.

**See above discussion about the use of the term “wildlife habitat”**

Line 445: See above, there is no such thing as wildlife habitat.

**See above discussion about the use of the term “wildlife habitat”**

Lines 482 – 496: Consider discussing the importance of disturbance for many species of grassland birds (Derner et al. 2009), and the potentially the importance of CRP different spatial scales.

**Thank you for this suggestion. We revised the paragraph to explicitly acknowledge the importance of disturbance and spatial scale in shaping CRP outcomes for grassland birds. We added references suggested by the reviewer, and included mention of mid-contract management practices as a tool to introduce disturbance and improve habitat suitability for disturbance-dependent species.**

Lines 493 – 494: Consider providing relevant references for Chestnut collared Longspur and Baird's Sparrow, or choose other species to discuss. The references cited are inappropriate for habitat relationships of these species on the above list. There is actually a fair bit of controversy on the importance of CRP to local habitat requirements for species such as Chestnut collared Longspur and Baird's Sparrow, and well as disturbance-dependent species (Derner et al. 2009). Many sources, including Shaffer et al. (2020), suggest most CRP provides poor habitat for Baird's Sparrow. The Chestnut collared Longspur often requires habitat conditions with greater disturbance than is afforded by CRP. Nevertheless, Pavlacky et al. (2022) found positive effects for Chestnut collared Longspur and Baird's Sparrow from large-scale connectivity of increasing CRP cover in the surrounding landscape.

This may also be a good place to discuss the importance of mid-contract management CRP Grasslands to introduce disturbance and provide suitable habitat for disturbance-dependent species.

**We appreciate this important clarification. In response, we revised the paragraph to note that CRP may not always provide suitable habitat at the site scale for disturbance-dependent species such as Baird’s Sparrow and Chestnut-collared Longspur. We also clarified the effects of CRP outcomes on disturbance-dependent species as described in the above response. The new paragraph section now reads: “Some of these species, particularly Field Sparrow, Henslow’s Sparrow, Lesser Prairie-Chicken, and Greater Prairie-Chicken are believed to have benefitted substantially from CRP, with steep declines through much of the 20th century appearing to have stabilized to some degree in part due to efforts to restore their habitat (Herkert 2007; Tanner et al. 2021). For other species, such as Baird’s Sparrow and Chestnut-collared Longspur, CRP may offer less suitable habitat at the site scale due to the absence of disturbance regimes (e.g., grazing, fire) that these species often require (Derner et al. 2009; Shaffer et al. 2020). Nevertheless, studies suggest that larger-scale connectivity provided by CRP cover in agricultural landscapes may still support positive outcomes for these disturbance-dependent bird species (Pavlacky et al. 2022). These examples underscore the importance of considering both spatial scale and management practices—such as mid-contract management techniques that reintroduce disturbance—when evaluating CRP’s effectiveness for grassland bird conservation. Additional research that includes other species, both avian as well as non-avian wildlife, and that considers the above-described types of region and species-specific responses to particular management approaches and types of disturbance, can help to bolster conservation efforts and add to the list of CRP success stories.” [Lines 510-525]**

Lines 498 – 532: Consider short paragraph to discuss the sensitivity of the conclusions to double counting studies in the summaries presented in the results section. This may be yanking around the conclusions based on summarizing the frequency of studies. Even a scoping review requires an evaluation that claims are supported by the data.

**Thank you for raising this point. We agree it is important to be transparent about how studies were categorized and counted. In the methods section, we revised to clarify that categories were not mutually exclusive and that some studies contributed to more than one group (see above response). Because our results are presented as the number of studies per category, and we do not sum across categories to report aggregate totals, this approach does not bias our conclusions. This type of overlapping classification is common and accepted in scoping review literature, particularly when studies span multiple relevant domains. Given this, and the existing clarification in the text, we did not add a full additional paragraph but are open to expanding further if needed.**

Lines 534 – 547: Consider discussing the importance of addressing information gaps to achieve multi-objective conservation planning to balance transdisciplinary outcomes.

**Thank you for this suggestion. We agree that addressing information gaps through research that integrates ecological, agricultural, and social outcomes is critical for informing multi-objective conservation planning. In response, we have added a sentence to the concluding paragraph that highlights the value of such transdisciplinary approaches. The revised text now includes: “In addition, future research that integrates ecological, agronomic, and socioeconomic outcomes will be especially valuable for informing multi-objective conservation planning.” [Lines 572-574]**

Line 537: See above, no such thing as wildlife habitat

**See above discussion about the use of the term “wildlife habitat”**

Literature Cited

Butsic, V., T. Kuemmerle, L. Pallud, K. J. Helmstedt, L. Macchi, and M. D. Potts. 2020. Aligning biodiversity conservation and agricultural production in heterogeneous landscapes. Ecological Applications 30:e02057.

Derner, J. D., W. K. Lauenroth, P. Stapp, and D. J. Augustine. 2009. Livestock as ecosystem engineers for grassland bird habitat in the western Great Plains of North America. Rangeland Ecology & Management 62:111-118.

Hall, L. S., P. R. Krausman, and M. L. Morrison. 1997. The habitat concept and a plea for standard terminology. Wildlife Society Bulletin 25:173-182.

Johnson, M. D. 2007. Measuring habitat quality: a review. The Condor 109:489-504.

Pavlacky, D. C., Jr., A. W. Green, T. L. George, R. Iovanna, A. M. Bartuszevige, M. D. Correll, A. O. Panjabi, and T. B. Ryder. 2022. Landscape-scale conservation mitigates the biodiversity loss of grassland birds. Ecological Applications 32:e2548.

Shaffer, J. A., L. D. Igl, D. H. Johnson, M. L. Sondreal, C. M. Goldade, M. P. Nenneman, and B. R. Euliss. 2020. The effects of management practices on grassland birds: Baird’s sparrow (Centronyx bairdii). Professional Paper 1842–HH. in D. H. Johnson, L. D. Igl, J. A. Shaffer, and J. P. DeLong, editors. The effects of management practices on grassland birds. Professional Paper 1842. United States Department of the Interior, U. S. Geological Survey, Reston, Virginia, USA. . Accessed 21 March 2022.
